# Prediction of compound-target interaction using several artificial intelligence algorithms and comparison with a consensus-based strategy

**DOI:** 10.1186/s13321-024-00816-1

**Published:** 2024-03-07

**Authors:** Karina Jimenes-Vargas, Alejandro Pazos, Cristian R. Munteanu, Yunierkis Perez-Castillo, Eduardo Tejera

**Affiliations:** 1 Bio-Cheminformatics Research Group, Universidad de Las Américas, Quito, 170504 Ecuador; 2 Departament of Computer Science and Information Technologies, Faculty of Computer Science, Universidade da Coruña, Campus Elviña s/n, 15071 A Coruña, Spain; 3 CITIC-Research Center of Information and Communication Technologies, Universidade da Coruña, 15071 A Coruña, Spain; 4 Biomedical Research Institute of A Coruña (INIBIC), University Hospital Complex of A Coruna (CHUAC), 15006 A Coruna, Spain

**Keywords:** Target identification, Target fishing, Ligan-based modeling, Machine learning, QSAR

## Abstract

For understanding a chemical compound’s mechanism of action and its side effects, as well as for drug discovery, it is crucial to predict its possible protein targets. This study examines 15 developed target-centric models (TCM) employing different molecular descriptions and machine learning algorithms. They were contrasted with 17 third-party models implemented as web tools (WTCM). In both sets of models, consensus strategies were implemented as potential improvement over individual predictions. The findings indicate that TCM reach f1-score values greater than 0.8. Comparing both approaches, the best TCM achieves values of 0.75, 0.61, 0.25 and 0.38 for true positive/negative rates (TPR, TNR) and false negative/positive rates (FNR, FPR); outperforming the best WTCM. Moreover, the consensus strategy proves to have the most relevant results in the top $20\%$ of target profiles. TCM consensus reach TPR and FNR values of 0.98 and 0; while on WTCM reach values of 0.75 and 0.24. The implemented computational tool with the TCM and their consensus strategy at: https://bioquimio.udla.edu.ec/tidentification01/. Scientific Contribution: We compare and discuss the performances of 17 public compound-target interaction prediction models and 15 new constructions. We also explore a compound-target interaction prioritization strategy using a consensus approach, and we analyzed the challenging involved in interactions modeling.

**Graphical Abstract:**

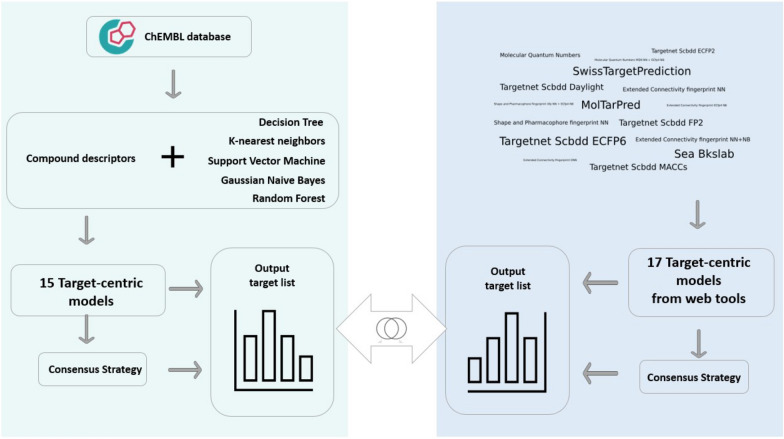

**Supplementary Information:**

The online version contains supplementary material available at 10.1186/s13321-024-00816-1.

## Introduction

Predicting protein-small molecule interactions is a problem at the core of drug discovery and system biology. This problem has traditionally been addressed using a variety of experimental techniques, including affinity chromatography, drug affinity determinations, responsive target stability, and others [1]. Nevertheless, these techniques are laborious, costly, and not suitable for large molecular screenings. As a result, computational approaches have also been considered plausible initial alternatives because they can be easily integrated with experimental validations to quickly narrow down potential targets to a small number of the most likely candidates [2].

The problem of compound-target interaction prediction can be related to several applications and has numerous alternate names across the literature, like target identification, in-silico target prediction, target fishing, or polypharmacology prediction [3–5]. Target identification (TI) consists of predicting a list of possible targets (target profile) for a *drug*/*compound* that are sorted by a *ranking*/*probability* criterion provided by statistical models [5]. In fact, the discovery of new targets may lead not only to a better understanding of efficacy, side effects, and mechanism of action but also to drug repositioning [6]. Therefore, this is a highly relevant problem for system biology, cheminformatics, and bioinformatics.

Traditionally, two main approaches are used to address compound-target interaction: ligand-based and target-based modeling. The ligand-based models are driven by the similarity principle and rely on the structural information and physicochemical properties of the known active and inactive compounds? scaffold [5]. In this approach the knowledge of the target protein structure (or even phenotype) is not required. The target-based models exploit the three-dimensional (3D) structure of the target [7] and the requirement of having a group of active and inactive molecules is not required. Molecular dynamics simulation and docking are commonly used in target-based modeling.

The ligand-based methods are generally faster and simpler compared to those used in structure-based models. In this sense, several reviews have been published about these strategies [8, 9]. The present work focuses on ligan-based modeling. The main purpose of TI is exploiting large groups of molecules with known biological activities to predict compounds' interaction for a particular target.

Molecules can be encoded by chemical descriptors (e.g., ECFP, MACCS), which are generally fast to compute. The advantage of using them over time-consuming techniques (conventional methods, such as experimental validation) is that predictions of new compounds can be made quickly [10]. After encoding the first stage, there are further steps to carry out the actual prediction. In this direction, a wide spectrum of strategies based on chemical similarity search, and/or machine learning (ML) are explored for TI in several published reports [5, 11]. The chemical space is huge, and only a small fraction of compounds are known [12]. Nonetheless, many TI models show good performance if robust databases are used for model construction and validation. This work focuses on ML methods.

The similarity search method has proven to be simple and fast because it only uses a *distance/similarity* metric of choice and an encoding for compounds [4]. A major limitation of this approach arises when novel query compounds have low similarity to those included in the training data because no similar compounds can be found on the reference datasets. Using queries from new chemical domains initially ignored by the models are responsible for performance decrements, but this is not the only factor. Identifying an appropriate similarity threshold to reduce the number of false positives remains an important challenge [13]. This similarity search is applied in different ways such as the targets annotated with top k-nearest neighbors of the query molecule [3], the targets at the top of the ranking list are arranged according to how similar the query is to each target’s three closest neighbors, on average [14]; and the targets at the highest-ranking ordered by statistic e-values [15].

The ML-based methods are more accurate but require a considerable amount of reliable data to fit a statistical model to quantify how chemical descriptors relate to activity [4], and have superior extrapolation capabilities to identify molecular targets compared to the similarity search, which utilizes the entire feature descriptors without feature selection [5]. ML, in this context, is more frequently used for classification than regression, combines different types of algorithms for finding patterns in different types of data, and comprises *Quantitative Structure-Activity Relationships* (QSAR) and *Proteochemetrics modeling* (PCM). QSAR learns from the ligands’ information and makes model decisions [16]. The PCM is seen as an extension of traditional QSAR because it considers both ligand and target spaces to extrapolate compound activity to targets and makes up for some important interactional information [17].

In recent studies, other perspectives, like deep learning, have been explored. For example, both molecular and target representations of curated industry-scale benchmark datasets are used to build a structure-aware graph neural network by combining predicted contact maps and graph neural networks, unlike ML methods, which pays more attention to molecular representation as described above [18, 19].

The compound-target interaction prediction problem had been addressed from a wide range of ligand-based models and many of these models can be used from public web-tools. However, in addition to the evaluation of the individual performance of these models, we need to explore if a consensus approach could improve the prediction. Consensus strategies have demonstrated that combining diverse models and methods can enhance the reliability of predictions in several topics like gene prioritization [20] and classification problems [21]. However, even when used in ligand-target prediction [22–25], no rigorous consensus evaluation had been done.

In this work, 15 models are trained across several targets using diverse chemical descriptions and ML strategies for TI. The data is retrieved from the ChEMBL database [26] for training models. These models are called target-centric models (TCM) to differentiate them from target-based models. They are constructed for each target but rely on ligan-based methods. The TCM are also compared with 17 state-of-the-art models available as web tools (WTCM) relying on ML and similarity searching methods. In addition, the potential benefits of consensus strategies for improving the predictive and ranking capabilities of individual models are explored for both groups (TCM and WTCM). Additionally, in order to improve the performance of individual models and expand the target space pattern, a consensus analysis is investigated as a potential tactic. The best combination of algorithms and molecular descriptions is discussed in the context of an individual model and a fusion strategy. Finally, a computational tool is implemented for TCM and their consensus strategy.

## Methods

A subset of compound-target interactions, restricted to *Homo Sapiens*, was extracted from the chemo-genomics database ChEMBL [26].

The releases 27 (351,778 compounds, 1448 targets, and 504,747 protein-compound interactions), 28 (377,936 compounds, 1562 targets, 542,790 compound-protein associations), and 31 (403,364 compounds, 1668 targets, 579,009 compound-protein pairs) were used to build the datasets for 1) data pre-processing, 2) TCM, 3) WTCM, and 4) consensus strategy.

### Data pre-processing

The curation of compound-protein interaction data is a complex process, especially if many associations are required for modeling. For all ChEMBL releases, the same filtering strategy was used for data cleaning, following four rules: 1) only assays reporting *IC*50 values were included; 2) all units were converted to $\mu M$; 3) if a target-compound pair appears in more than one assay, then the median absolute deviation was computed to be used for outlier detection as proposed in [27] and the final median was assigned as the *IC*50 value for the interaction; 4) all interactions with $IC50 <= 10 \mu M$ were classified as active associations, and those with values $IC50 > 10 \mu M$ as inactive. The $10 \mu M$ cutoff is typically used in several studies [13, 28] to establish an association as active. It carries a significant influence on the effectiveness and applicability of target prediction methods [4], and in an experimental context, makes the most efficient use of costly experimental validation according to [29].

After applying this filtering approach to all three releases, a total of 350,818 compounds, 1521 targets, and 507,553 compound-protein pairs common to database releases 27 and 28 were taken for training TCM (the initial quantities after preprocessing of each release is presented in Table 1). The external dataset comprises all associations found in release 31 and not in the other two. A total of 52,874 unique molecules fulfill this criterion, covering 1196 targets and 74,987 molecule-target associations for external validation (see the summarized data curation process in Additional file 1: SM1.1, SM1.2 and Fig. SM1.1). Only the unique compound-target interactions of the releases 27 and 28 were used to define the TCM models. The other interactions that only appear in 31 and not in 27 and 28 were used later to evaluate both groups of TCM and WTCM models (see detail in Additional file 1: SM1.2)

At this point, two datasets were formed for training and for external validation, but further processing was required to have consistent data. In this sense, a minimum number of 10 active + 10 inactive compound-target interactions were required for training each target model in both datasets. Moreover, only targets present in both datasets and having no less than five interacting compounds per class (active or inactive) were considered for external validation, as suggested in [30]. In consequence, the training dataset was reduced to 253 targets, 184,046 compounds, and 249,269 interactions (labeled as DS1, see Table 1, Additional file 1: SM1.1 and SM1.2). The resulting external dataset (labeled as VDS2) contained 253 targets, 30,526 compounds, and 42,382 interactions.

**Table 1:** Datasets creation from ChEMBL database

ChEMBL release	Dataset	Process	Compounds	Targets	Interactions
27	CH27	Cleaning	351778	1448	504747
28	CH28	Cleaning	377936	1562	542790
31	CH31	Cleaning	403364	1668	579009
27 & 28	DS1	Training	184046	253^1^	249269
31	VSD2	External validation	30526	253^2^	42382^3^
31	VSD3	Contrast groups	3264	126	4716^3^

^1^ Target has at least 10 chemical interactions, both active and inactive. ^2^ Target has at least 5 chemical interactions, both active and inactive. ^3^ These compound-interactions are only in ChEMBL release 31 but not in 27 or 28

Then, for evaluating the web tool models, the initial idea was to predict the whole VDS2 set (30,526 compounds) with both TCM and WTCM. However, some web tools were slow, had limitations with the number of compounds that they could process and manifested some overflow problems. Also, it was crucial to note that it was impossible to define the full common target space between them (not all the web tools report the full list of targets on which a prediction can be performed and the TCM’ target profile is limited by the available data). Because of the above limitations, to compare the performance of both sets of models and their consensus strategy, after considering the applicability domain of TCM, a sub-sample of 3264 molecules from VDS2 was taken into account. This last dataset was labeled as VDS3, and its molecules represent interactions with 126 targets (out of the 253 used for TCM).

### Target-centric models

For each target in DS1, TCM models were trained considering the following process (for a more detailed description see Additional file 1: SM1, Note SM1.2). First, three different molecular representations: (1) 1024 bits of Morgan's fingerprint with a radius of eight (FGP); (2) 123 general molecular properties (DSC); and (3) The union of both FGP and DSC (FUS). Also, to deal with the unbalanced distribution of classes for training, a clustering strategy is applied for all molecules in each group of descriptors. A random sampling is carried out in each cluster to obtain a representative set of the majority class that is equal to the minority one. It indicates that the models are constructed using the same data in each group of descriptors (the same random split), but not across all description spaces. This was done to possibly improve further consensus strategy more than focused on the comparison across different descriptions.

TI was conceived as a classification problem and there is limited information from a compound about complex interactions for most of the targets, so each TCM was built for each target with common ML algorithms: Decision Tree (DT), Random Forest (RF), K-nearest neighbors (KNN), Support Vector Machine (SVM), and Gaussian Naive Bayes (GM). A total of 15 TCM (five ML algorithms and three molecular representations) were computed without any feature filtering or selection. The random $30\%$ of the total data (DS1) was used for evaluation (in addition to VSD2).

The applicability domain(AD) of each TCM was also determined to represent the region in space where the compounds were located [31]. The AD was defined using a distance-based method using the hamming distance for FGP, the euclidean distance for DSC, and both were applied simultaneously for FUS (see also Additional file 1: Note SM1.2.). The AD was validated for each compound in VSD2 before addressing the model and evaluating it with the f1-score. The most sensitive score to data distribution used on unbalanced is the f1-score [32], which is the harmonic mean of precision and recall.

### Target-centric models from web tools

A collection of 17 publicly accessible WTCM models that might be used as web tools are used for benchmarking. *MolTarPred* [33],*SwissTargetPrediction* [34],*TargetNet* [35], *Sea Bkslab* [35], *Sea Bkslab* [36], and *PPB2* [37] exploited different prediction strategies with six different fingerprints and the fusion of them. A more detailed description of each web-service is provided in Additional file 1: SM1.3.

Compounds in VSD3 were used as input to WTCM algorithms using scraping strategies. The target space (see details in Additional file 1: Table SM1.2) is different for each model prediction. So, for a particular method (compound representation + ML algorithm) the metrics of true positive rate (TPR), false positive rate (FPR), true negative rate (TNR), and false negative rate (FNR) were computed to evaluate and compared the WTCM with the 15 TCM. These measures work well with unbalanced data since they are not affected by shifts in the distribution of the data [38]. However, the implications of unbalanced validation data will be discussed in future sections.

Furthermore, the recovery rate and unknown rate measures were developed for each compound because the entire target space of each model is not always known and because the space varies among models. The recovery rate measures the fraction of all targets present in the ChEMBL database for which a prediction can be made by a specific method. On the other hand, the unknown rate represents the proportion of interactions predicted by a method for a given compound that has no experimental information in ChEMBL to be assessed. A more detailed description is presented in Additional file 1: SM1.3.

### Consensus approach

Each WTCM had a particular way of ranking the target profile, its prediction scores had different scales, and the target list size was different. Hence, to integrate all their predictions into a consensus score, some transformations were made to have all the scores on the same scale. The consensus score for the compound-target interaction was computed to get a value [0, 1] as a ranking criterion (a detailed description of this process is presented in Additional file 1: SM1.4). Then, a threshold of 0.5 was proposed to classify active or negative interactions with consensus. Next, targets were sorted descending by the consensus and several top-ranked fractions from $1\%$ to $100\%$ (step size of $5\%$) were defined for evaluating the performance of each fraction in terms of TPR, TNR, FPR, FNR, recovery, and unknown rate.

In the case of TCM, the predicted target lists per compound were the same size regardless of the employed modeling method. Therefore, an analysis was done to keep a representation of the 15 models and to maintain their diversity before computing the consensus score in VSD3. For this, a similarity matrix was built to create a hierarchical clustering in VSD2, and three cutoffs were set to identify and evaluate three representative groups. Then, the consensus score was also calculated for the group with the best performance in VDS3. The mean across all prediction probabilities were computed, and the target profile was sorted. Then, different top-ranked subsets from $1\%$ to $100\%$ (step size of $5\%$) were also defined to be evaluated with the metrics of TPR, TNR, FPR, FNR, recovery, and unknown rate.

## Results

### Target-centric models

**Fig. 1:**
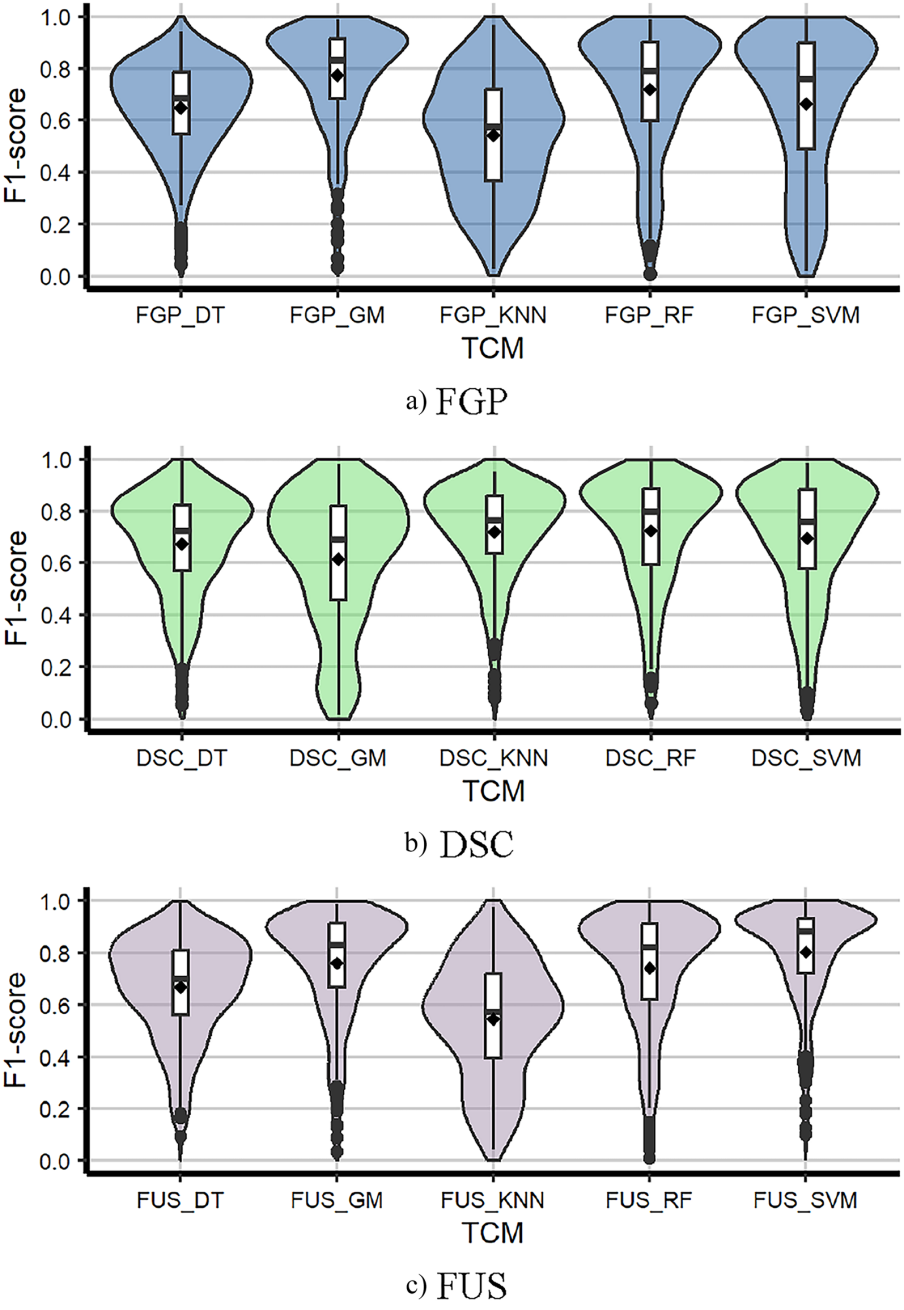
Performance target-centric models (TCM) on VDS2. TCM were trained with decision tree (DT), gaussian naive bayes (GM), k-nearest neighbors (KNN), random forest (RF) and support vector machine (SVM). The descriptors used were: **a** morgan's fingerprint (FGP), **b** molecular properties (DSC) and **c** the fusion of FGP and DSC (FUS)

The results of the 15 TCM are shown in terms of the f1-score in Fig. 1 for the 253 targets in VSD2. The remaining performance metrics are given as Additional file 2: SM2 in Table SM2.1-SM2.4. Besides, the average performance of each model across all targets using $30\%$ of the data in DS1 for testing is detailed in Additional file 2: Table SM2.1. The stability and variations of the performance metrics in Additional file 2: Table SM2.1 suggest that in general the obtained models are not overfitted.

According to the findings, the FGP$\_$GM, FGP$\_$RF, and FGP$\_$SVM models obtained f1-scores of $0.77\pm 0.19$, $0.71\pm 0.23$, and $0.66\pm 0.27$, respectively, in *FGP*. In contrast, *DSC* achieves the best performance when the DSC$\_$RF, DSC$\_$KNN, DSC$\_$SVM, and DSC$\_$DT are used, achieving f1-scores of $0.72\pm 0.21$, $0.71\pm 0.18$, $0.69\pm 0.22$, and $0.67\pm 0.2$. In several cases, the fusion of several descriptors increases the amount of information used to codify chemical structures, and consequently the ML performance by allowing the discrimination of small chemical differences. FUS descriptors produce f1-scores that are slightly higher than those produced by FGP and DSC. The best f1-scores obtained are $0.8\pm 0.19$ (FUS$\_$SVM), $0.76\pm 0.21$ (FUS$\_$GM), $0.74\pm 0.23$ (FUS$\_$RF), and $0.67\pm 0.19$ (FUS$\_$DT). The best score achieved is a f1-score of 0.8 with FUS$\_$SVM, which is slightly better than for other models like FGP$\_$GM, FUS$\_$GM, and FUS$\_$RF.

As previously described in the methodology section is not strictly convenient to compare across description spaces but between models with the same description because the dataset is not the same after random partition (intended to improve consensus approach). However, applying a statistical analysis of the metrics suggests statistically different distribution even across some models using the same description space ($p-value = 2.2e-16$, one way ANOVA). Also, the pairwise comparison is reported in (see Additional file 2: Table SM2.5, in SM2).

It is also clear that KNN outperforms FGP and FUS when using DSC. This can be because KNN uses a simple strategy for learning and might not handle higher dimensionality due to the presence of irrelevant and redundant attributes [39]. It appears that the vectors of FGP (1024 size) and FUS (1147 size) are not sufficiently short for using this learning model. The feature dimension reduction before training each TCM is one potential solution to this issue, but this process has certain drawbacks, such as data loss, computationally demanding procedure, most existing approaches are based on unrealistic assumptions of the underlying data structure, and converted features are often difficult to interpret [40].

The remaining TCM developed provide satisfactory results using the three FGP, DSC, and FUS investigated characteristics, suggesting that SVM, GM, RF and DT can learn patterns with any type of data. Further, RF learning appears superior with FUS than with FGP and DSC descriptors due to their capability to work with higher dimensionality and mixed types of data. RF can handle binary, category, count, and continuous variables since they only use a portion of the independent variables rather than dealing directly with all of them at once [41].

If the f1-scores obtained from the random split of $30\%$ are compared with the values obtained from VDS2 (Additional file 2: SM2.1 and SM2.3). It can be noticed that in general a reduction of the f1-score but a more drastic change in other metrics like accuracy or specificity. For example, the FGP$\_$GM shown an f1-score, specificity and accuracy of 0.77, 0.26 and 0.69 in VSD2 compared to 0.83, 0.79 and 0.83 with the random split of $30\%$. The reduction of specificity is constant across all models with all descriptors. These can be due two main factors responsible for this observation: (1) The molecular diversity is higher in VSD2 compared to the $30\%$ and (2) The unbalanced distribution of the active/inactive classes in the VSD2 validation dataset. The average distance in DS1 from all compounds to the centroid in each target is lower than those taking part in VDS2 (see Additional file 2: Table SM2.4 and Fig. SM2.1). Therefore, the modification in the performance is more likely to be related with the fact that in VDS2 the information from the new targets interactions is mostly unbalanced toward the active class.

The unbalanced nature of the active class will directly affect the TP and FN and consequently affect performance. In this case, the values of precision and specificity tend to be higher than recall in Additional file 2: SM2.1 (random partition of the $30\%$ in DS1) therefore our models will be specially affected from an unbalanced external data towards the active class. A possible solution will be the reduction in the number of actives in the external or to carried out a data augmentation of the inactive class. However, by reducing the active class we can lose some targets or even overestimate the performance. The augmentation using molecular decoys is worthy worth exploring in future works.In further studies, it could be worthwhile to investigate the augmentation technique utilizing molecular decoys.

### Comparison of TCM with the WTCM

**Fig. 2:**
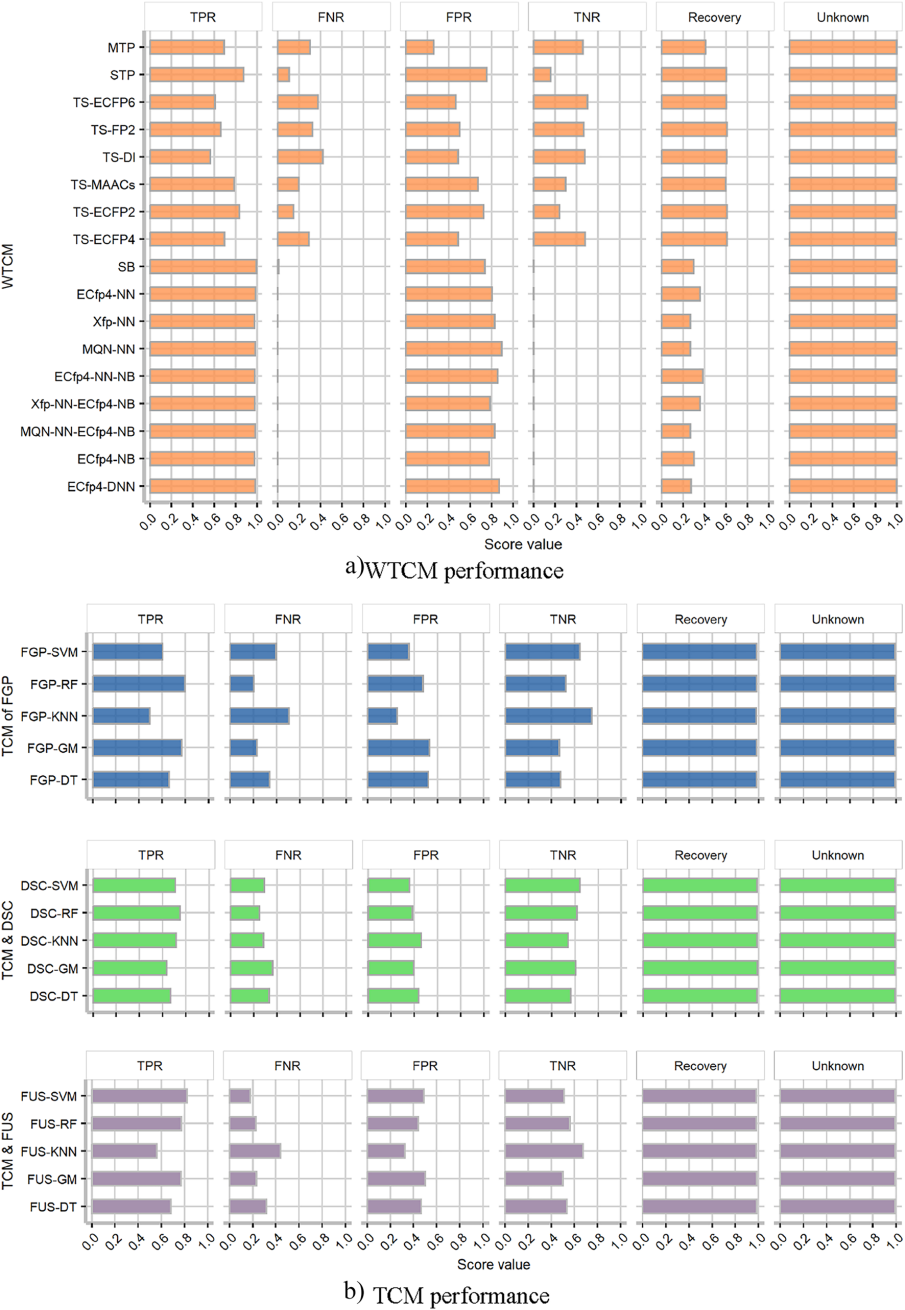
Comparison of **a** target-centric models from the web tools (WTCM) and **b** target-centric models (TCM). TCM were trained with morgan's fingerprint (FGP), molecular properties (DSC) and the fusion of both (FUS) descriptors; and the algorithms of decision tree (DT), gaussian naive bayes (GM), k-nearest neighbors (KNN), random forest (RF) and support vector machine (SVM)

The WTCM and TCM are evaluated using VSD3, which encompasses a group of 3264 compounds and 126 targets. These compounds are within the AD of each trained TCM and contain information about their interactions with at least one of the 126 targets. Figure 2 illustrates the performance of the WTCM and the TCM in terms of TPR, TNR, FPR, and FNR with VDS3 (see values in Additional file 2: Table SM2.5 and Table SM2.6).

According to the results of the WTCM, the models MTP, TS-ECFP6, and ECfp4-NN have good performance metrics. MTP has TPR, TNR, FPR, and FNR values of 0.69, 0.45, 0.26, and 0.29, respectively, while TS-ECFP6 and TS-ECFP4 achieve similar results (TS-ECFP4 showed a small increment in TPR with respect to MTP and TS-ECFP6). Additionally, the last 8 algorithms (Fig. 2a) only provide positive interaction predictions; therefore, it is not possible to compute the TNR and FNR in those cases. The results of these last algorithms (SB, ECf4-NN, Xfp-NN, MQN-NN, ECf4-NN-NB, Xfp-NN-ECfp4-NB, MQN-NN-ECfp4-NB, ECfp4-NB, ECfp4-DNN) shows that the performance is not better than the other algorithms from web tools because even though the TPR values are over 0.98; the FPR values achieved are higher than 0.74. The findings also indicate that these models’ recovery rates range from 0.3 to 0.6, whereas the unknown rates have a high value of 0.99.

The models FGP$\_$SVM, DSC$\_$GM, DSC$\_$RF, and DSC$\_$SVM from the TCM have a good performance as shown in Fig. 2b. These models achieve TPR and TNR values over 0.6, while the FNR and FPR metrics are among the lowest (best), with values around 0.3 (which is consistent with the previous result during validation with the VDS2 dataset). The recovery rate and the unknown rate have a score of 0.98.

In general, TCM models seem to perform better than WTCM due to the higher TPR and TNR values (over 0.6) as well as the lowest FNR and FPR values (under 0.3). Although, TCM models have higher values for the recovery rate than WTCM, the values of unknown rates are high in both TCM and WTCM. Most of the recovery values in TCM are over 0.9, and the models from WTCM achieved the highest scores (over 0.8) with TS-FP2, TS-Daylight, TS-MAACs, TS-ECFP2, and TS-ECFP4; while the other WTCM reached values below 0.4. The higher the recovery rate, the more accurately the key prediction performance indicators can be estimated, which means, the performance of WTCM could be overestimated due to a lack of information.

In Fig. 2, the average metrics are presented, however, in Additional file 2 (SM2) the standard deviation was added. The standard deviation should be analyzed carefully because in some models we don’t know the negative predictions (it is not possible to compute f1-score) and the target space across all studied models is not the same and in some cases it is unknown. These reasons lead us to present the recovery and the unknown metrics in our analysis. In this context, a key challenge in validating compound-target interaction predictions, especially for multiple targets, is the lack of information. In many cases, there is no information in the dataset to establish whether the predicted interaction is correct or not. Most molecules in ChEMBL only have reported interactions (active or inactive) with one or two targets on average [42, 43]; therefore, it is likely that many predictions will not have experimental evidence to be validated.

The computation of the evaluation metrics is performed in a fairly limited space of predictions in which there is information to validate. Regarding the unknown values, which are around 0.9 in both TCM and WTCM, the highest portion of the predictions do not have experimental information for validation. This could be a plausible explanation for the low global performance values of any model. Let’s examine two examples to illustrate this issue.

1. For each compound, a total of 126 predictions could be made (there are 126 targets in VSD3). If a compound has 12 predictions with different targets registered in ChEMBL, and only 9 TCM exist out of the total 12. The performance metrics can be computed for the predictions for 9 targets, and the remaining 117 targets cannot be validated due to a lack of information. Therefore, the compound is validated with an output confusion matrix given by $TP=2$, $FP=2$, $TN=2$ and $FP=1$ in which the remaining $unknown=2$ is undetermined because the compound is outside the AD of the 9 TCM. The resulting recovery rate is 0.78 (7/9) and the unknown rate is 0.94 $(126-7)/126$).
2. The previous example is even more complicated for average performance metrics. It means, using a single algorithm (e.g. FGP$\_$SVM) the minimal number of molecules and targets to evaluate are $10*126$ (because 5 inactive/active predictions were considered as minimum requirements for each target). Therefore, using one algorithm, a total of $10*126*126 = 158 760$ predictions can be performed if the compound is inside the AD of the TCM. All molecules from each target could be predicted against all possible targets. If the maximal number of targets commonly found in the database for a particular compound is two, only $10*126*2 = 2520$ predictions out of 158,760 (around $1.6\%$) can be used to evaluate the model. This number is around the observed values in unknown rate.

This amount of information is very low, and therefore the performance metrics obtained here (and in any other possible model computed with similar approaches) could not represent the actual model performance. However, considering the performance metrics obtained with *DS*1 and* VSD*2 (which are higher and comprise more targets and compounds for validation), we think that the obtained metrics with TCM and WTCM using VDS3 tend to be underestimated. Even with this limitation, the main goal of VDS3 is to create a common ground for comparison between different models.

Our analysis intended to compare large number of models across different authors, they are constructed using different datasets and target spaces, which revealed to be a complex scenario for benchmarking. The different protein spaces and the different outputs from the WTCM and TCM make it difficult for any “meta-analysis” approach. However, it is easy to notice that the target space predicted is largest than the available experimental information needed to validate (higher values of unknown rate).

### Consensus approach

The target space is diverse as discussed previously, consequently a consensus approach could help to comprise more targets information across different models. The consensus approach is intended to evaluate if the combination of models provides better performance than single ones. The process considers the consensus of the WTCM and TCM group of models separately considering VSD3. Because different metrics are involved in each model, the main gol of consensus is to prioritize possible targets. In this sense some kind of *“ranking”* is necessary.

Before performing the consensus strategy with TCM models, a group of its most representative models is determined. A hierarchical clustering dendrogram is generated based on a similarity matrix and using target profile predictions on VSD2 (see details in Additional file 2, Note SM2.2). Three cutoff values (0.72, 0.75, and 0.8) are used to create clusters of 3, 5, and 7 TCM, respectively. The model with the highest f1-score is selected from each cluster to perform the ensemble fusion strategy.

Then, the best TCM (FUS$\_$SVM), the first cluster of 3 TCM (FUS$\_$GM, FUS$\_$RF, FUS$\_$SVM), the second one of 4 TCM (FUS$\_$GM, FUS$\_$RF, FUS$\_$SVM, FGP$\_$RF, DSC$\_$RF ), and the third cluster of 7 TMC (FUS$\_$SVM, FUS$\_$RF, FGP$\_$RF, DSC$\_$RF, FGP$\_$GM, DSC$\_$KNN, FUS$\_$GMM) were analyzed in terms of TPR, FNR, and FPR and TNR metrics over VSD2. Results indicate a small increment in the performance with 3 TCM. Also, regarding this clustering evaluation, ANOVA tests suggest statistically significant differences (p-values = 0.036).

**Fig. 3:**
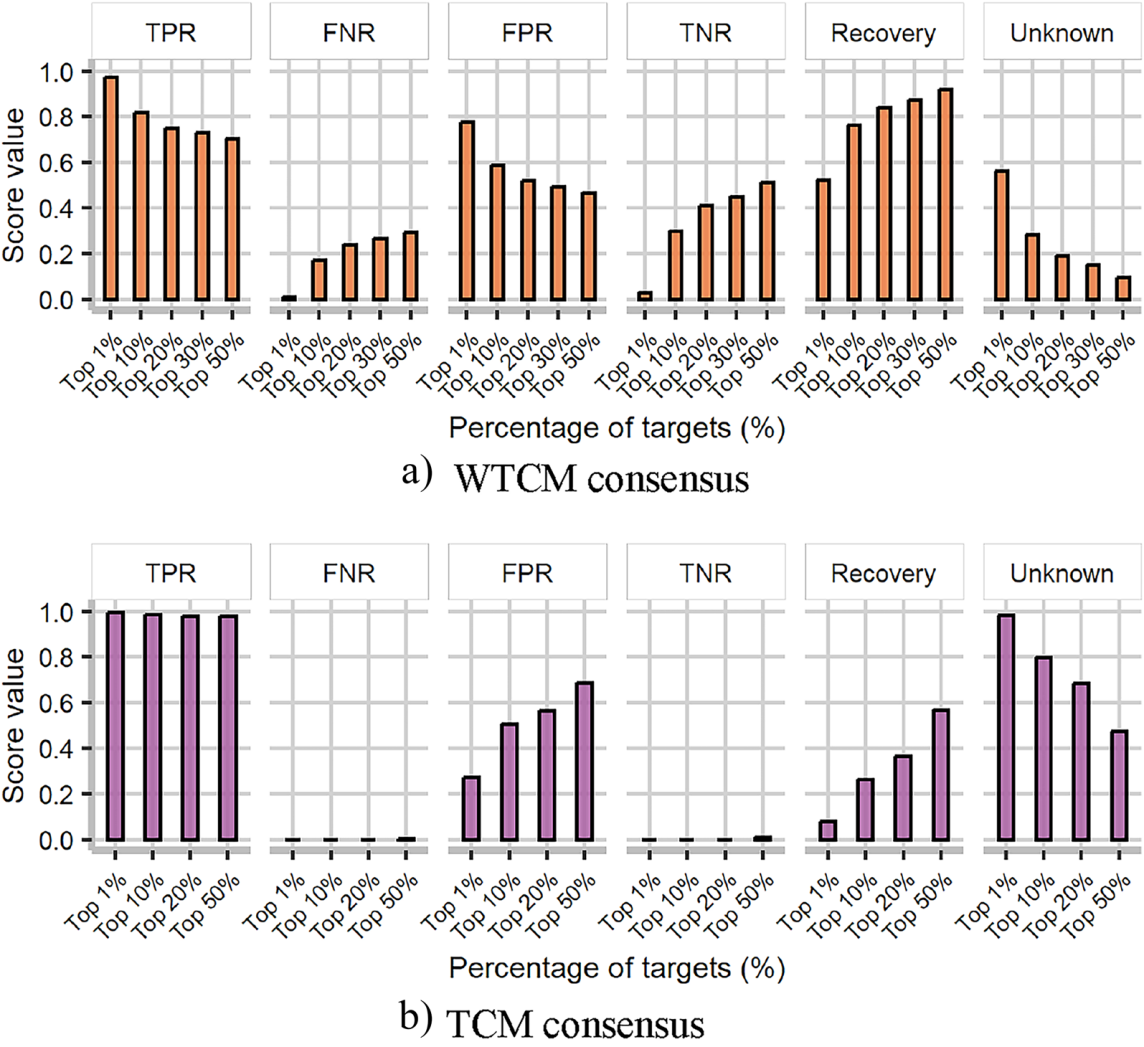
Consensus performance across the top percentage splits of the target profile for **a** target-centric models of the web tools (WTCM) and **b** target-centric models (TCM)

Based on the simplicity for the TCM consensus strategy as simple as possible, the final assembly comprises 3 TCM over VSD3 (which improves TNR, see Additional file 2: Figure SM2.1b). As previously discussed, in these metrics, only a small part of the predictions can be assessed. Thus, the consensus tries to establish a prioritization scheme. For a compound, the target profile is ranked according to the probability of being classified as active, and the performance metrics are computed at different splits of the ranked list. The results of this TCM consensus strategy analysis over VSD3 are presented in Fig. 3b and in Additional file 2: Table SM2.8.

Similarly, the consensus strategy is also executed over the WTCM and includes all models. Uniting all the models allows for the greatest amount of information regarding compound-protein interactions. This is important because the number of targets predicted by each model is different and the results depend on several external factors (sometimes there is no information, or the web tool is down). The algorithms were trained with different databases. They have different target profile output sizes and some of their models have been modeled using similarity search or ML techniques (see detail in Additional file 1: SM1.2). Also, since some of the algorithms only report positive interactions, negative interactions can not be inspected in some cases. The results of the WTCM consensus over VSD3 are illustrated in Fig. 3a (see values in Additional file 2: Table SM2.9).

Results suggest that the TCM consensus achieves much better TPR results than the WTCM consensus (Fig. 3); even though the target profile is bigger in the WTCM. The consensus over the TCM shows that the top $20\%$ (around the top 20 predicted targets) keep the TPR values above 0.9 while the TNR is 0. The FNR is also 0, the FPR is 0.56, the recovery rate is 0.37, and the unknown rate is 0.68. The TNR and FNR are lower or close to zero because all molecules at the top (less than $50\%$) will be classified as active. The top $20\%$ appears to retain the targets that are most indicative of the profile target and contain $42.4\%$ of the total targets.

In contrast, with $87.5\%$ of the total targets, the WTCM consensus appears to perform worse in the top $20\%$. The TPR and TNR scores are 0.75 and 0.41, respectively, and the FNR and FPR are 0.24 and 0.52. Also, the recovery rate is high and the number of unknown interactions is low (0.84 and 0.19). Another interesting result is that the recovery rate of each WTCM model is quite low (Fig. 2a), but this recovery considerably increases with the WTCM consensus approach (Fig. 3a).

Results also suggest that, if all targets for a particular compound are predicted and ranked by the probability of being active, the TCM will have more predictions ranked at the top with interactions known by the ChEMBL compared to WTCM. Moreover, among the top targets (less than $50\%$) found in TCM, almost all fall into active interactions, with fewer negative interactions than with WTCM. Although it is not possible (due to a lack of information) to evaluate all interaction predictions reported by WTCM, TCM provides a reliable set of new predictions. Moreover, in general, the predictions of TCM are better than those of WTCM, probably because the database releases used for training are updated. Consequently, the target space and molecular diversity considered during modeling are probably higher, allowing better generalization.

### Web-tool implementation

A freely accessible web tool without login credentials is created for performing TI with the 15 TCM and their consensus approach at https://bioquimio.udla.edu.ec/tidentification01/. It has a simple and intuitive interface with an input field for smiles of query compounds and four tabs for descriptors FGP, DSC, FUS, and the consensus approach. Besides, an example and a help section are included to make its use simpler and to explain how to interpret those results. More information regarding this web tool and its use is presented in Additional file 2: Note SM2.2.

## Conclusion

The TCM have good performance since the f1-score values reached values greater than 0.8. When comparing the FGP and DSC descriptors, the results indicate that the FGP perform better than the DSC. In this comparison, the highest scoring models are FGP$\_$GM and DSC$\_$DT with f1-score values of 0.83 and 0.79 respectively. Likewise, combining these two groups of descriptors in FUS to merge the available information improved the reliability of the model’s performance, the best models are FUS$\_$SVM with a f1-score of 0.88, FUS$\_$GM of 0.83, and FUS$\_$RF of 0.82.

The TCM produces good results compared to WTCM individual evaluations. Algorithms like FGP$\_$SVM, DSC$\_$GM, DSC$\_$RF, and DSC$\_$SVM perform better than the models evaluated in WTCM. Even though all TCM and WTCM have unknown values around 0.9. In comparison to the algorithms from web tools, the TCM’s recovery rates are higher with values over 0.9.

The consensus approach improves the performance of the individual TCM and WTCM. The consensus over TCM shows a little increment in performance with 3 of the 15 models, which allows to enhance results faster; as well as, the consensus over WTCM with all algorithms, which allows to increase the recovery rate even though the data collection process was slow. The evaluation of the interaction space is limited to the reduced amount of information. Even so, results suggest that the most representative interactions are found in the top $20\%$ in both approaches, and most of them are positive interactions. There is high confidence in this split when the TPR and FNR are above 0.9 and 0 in the TCM consensus and when they are around 0.75 and 0.23 in the WTCM consensus. It also shows that the recovery rate considerably increases in the consensus scores in contrast to the individual models.

### Supplementary information

**Additional file 1.** Extended methodology. Datasets. Target-centric Models (TCM). Target-centric Models from Web Tools (WTCM). Consensus strategies

**Additional file 2.** Extended results. TCM. Comparison between TCM and WTCM. TCM. Consensus approach. Web-tool implementation.

## Data Availability

The datasets and the trained target-centric models (TCM) are publicly available at the github repository at https://github.com/kbjimenes/target-identification.

## Abbreviations

TCM: Target centric model
WTCM: Target centric model from web tools
TI: Target identification
ML: Machine learning
QSAR: Quantitative structure-activity relationships
PCM: Proteochemetrics modeling
ChEMBL: Publicly available chemogenomics database.
FGP: Morgan’s fingerprint
DSC: Generals, structure and topological descriptors
FUS: The fusion of both FGP and DSC
DT: Decision tree
RF: Random forest
KNN: K-nearest neighbors
SVM: Support vector machines
GM: Gaussian naive bayes
AD: Applicability domain
TP: True positive
FP: False positive
TN: True negative
FN: False negative
TPR: True positive rate
FPR: False positive rate
TNR: True negative rate
FNR: False negative rate
