# Supplementary material for: Prediction of compound-target interaction using several artificial intelligence algorithms and comparison with a consensus-based strategy
Source: J Cheminform. 2024 Mar 7;16:27. doi: 10.1186/s13321-024-00816-1 (PMC10919000; doi:10.1186/s13321-024-00816-1)
Supplement: Supplementary file 1 — Additional file 1. Extended methodology. Datasets. Target-centric Models (TCM). Target-centric Models from Web Tools (WTCM). Consensus strategies [file 13321_2024_816_MOESM1_ESM.pdf]

Supplementary Material 1

SM1. Extended Methodology

Note SM1.1. Datasets

The *Homo Sapiens*-specific datasets of compound-target interactions were taken from the ChEMBL [1] database, taking into account three releases: 27 (351778 compounds, 1448 targets, and 504747 protein-compound interactions), 28 (377936 compounds, 1562 targets, 542790 compound-protein associations), and 31 (403364 compounds, 1668 targets, 579009 compound-protein pairs). ChEMBL is updated regularly and will eventually contain a deluge of fresh information that is being gleaned from scientific literature and patents [2].

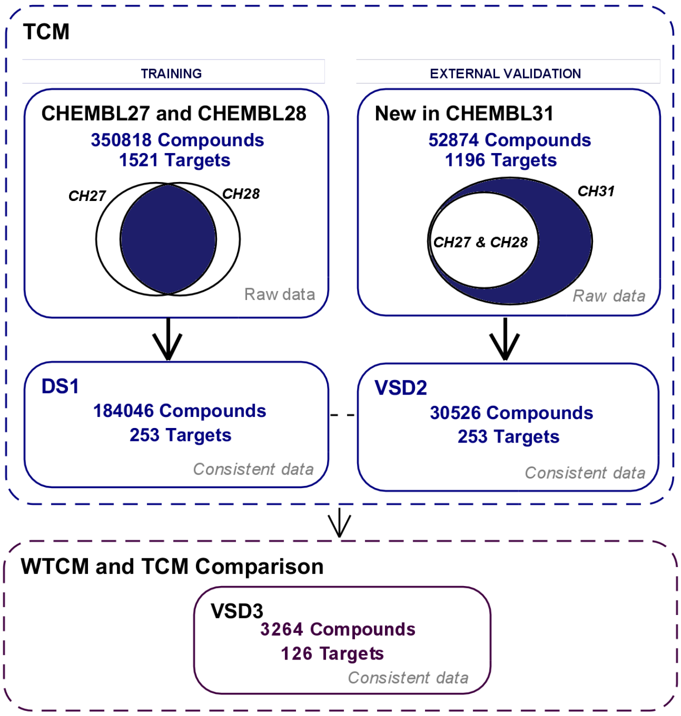

Fig. SM1.1 Dataset creation for training target-centric modeling (TCM) and for comparing them with models from web tools (WTCM).

All raw data from the three datasets releases (27, 28, and 31) were curated to ensure it is of high quality for the experiments. After that, a total of 350818 compounds, 1521 targets, and 507553 compound-protein interactions common to database releases 27 and 28 were taken for training target-centric models (TCM). The remaining compound-target associations found in release 31 and not in the other two (27 and 28) were considered for external validation(52874 compounds, 1196 targets, and 74987 molecule-target associations). Additionally, a minimum of 5 active/inactive compounds in both datasets was assessed to have consistent data. Therefore, the dataset for training TCM was reduced to DS1 (253 targets, 184046 compounds, and 249269 interactions). This also reduced the dataset for external validation which was labeled as VSD2 (253 targets, 30526 compounds, and 42382 interactions). The full data curation process was summarized in Figure SM1.1.

Moreover, for evaluating the target-centric models from the web tools (WTCM)and for comparing them with TCM and their consensus strategy, the dataset VSD3 was created. This is because, even though the initial idea was to use VDS2, it was not possible to take all the compounds from VSD2 and performs a prediction with the WTCM due to several external factors (unavailability of service, limited information, slow response times among others) from the web tools. Therefore, just a sample of 3264 molecules was taken into account.

Note SM1.2. Target-centric Models (TCM)

For each target in DS1, independently of the total of positive and negative interactions with compounds, individual target-centric models (TCM) were trained using three different molecular representations as features: 1) 1024 bits of Morgan's fingerprint with a radius of eight (FGP); 2) a set of 123 general physiochemical, structural, and topological molecular properties (DSC) (see Table SM1.1); and 3) The union of both FGP and DSC descriptors (FUS). The set of binary digits (bits) called FGPs represents the presence or absence of structural features in the molecule, where atom environments are included [3]. All molecular descriptors and fingerprints used in this study were computed with an open-source cheminformatics software RDKit[1] through its Python implementation [4].

Since Target identification was conceived as a classification problem and there is limited information from a compound about complex interactions for most of the targets, in this work, a TCM was built for each target with common ML algorithms instead of sophisticated algorithms like neural networks. The models were trained with the following five popular ML algorithms:

- Decision Tree (DT): uses a set of rules to make decisions, much like humans make decisions [5]. It employs the features to create *yes/no* questions and to split the dataset until the compounds are isolated into a particular class.
- Random Forest (RF): employs averaging to increase predictive accuracy and reduce overfitting by fitting several decision tree classifiers to different subsamples of the

[1] RDKit (https://www.rdkit.org/) is an Open-Source Cheminformatics Software. It has a collection of cheminformatics and machine-learning software written in C++ and Python

Table SM1.1 General physiochemical properties of interest.

Acronym	Description
MolWt	The average molecular weight of the molecule
ExactMolWt	The exact molecular weight of the molecule
HeavyAtomMolW	The average molecular weight of the molecule ignoring hydrogens
LogP	Wildman-Crippen LogP value
MolMR	Wildman-Crippen MR value
HAcceptors	The number of Hydrogen Bond Acceptors
HDonors	The number of Hydrogen Bond Donors
ValenceElectrons	The number of valence electrons the molecule has.
TPSA	The polar surface area of a molecule based upon fragments.
RotatableBonds	The number of Rotatable Bonds
LabuteASA	The Labute ASA value for a molecule.
AmideBonds	The number of amide bonds in a molecule.
Heteroatoms	The number of heteroatoms for a molecule.
SpiroAtoms	The number of spiro atoms (atoms shared between rings that share exactly one atom).
BridgeheadAtoms	The number of bridgehead atoms (atoms shared between rings that share at least two bonds).
FractionCSP3	The fraction of C atoms that are SP3 hybridized.
AromaticRings	The number of aromatic rings for a molecule.
SaturatedRings	The number of saturated rings for a molecule.
AliphaticRings	The number of aliphatic (containing at least one non-aromatic bond) rings for a molecule.
AromaticCarbocycles	The number of aromatic carbocycles for a molecule.
SaturatedCarbocycles	The number of saturated carbocycles for a molecule.
AliphaticCarbocycles	The number of aliphatic (containing at least one non-aromatic bond) carbocycles for a molecule.
AromaticHeterocycles	The number of aromatic heterocycles for a molecule.
SaturatedHeterocycles	The number of saturated heterocycles for a molecule.
AliphaticHeterocycles	The number of aliphatic (containing at least one non-aromatic bond) heterocycles for a molecule.
NumRings	The number of rings for a molecule.
CalcAUTOCORR2D	Returns 2D Autocorrelation descriptors vector.
BertzCT	A topological index meant to quantify "complexity" of molecules.
Ipc	The information content of the coefficients of the characteristic polynomial of a hydrogen-suppressed graph of a molecule.
HallKierAlpha	The Hall-Kier alpha value for a molecule.
CalcAUTOCORR2D	Returns 2D Autocorrelation descriptors vector.
Kappa[1-3]	Kappa Shape Indices.
Chi[0,1]	Atomic connectivity index [0,1].
Chi[0-4]n	Atomic valence connectivity index [0-4].
Chi[0-4]v	Atomic valence connectivity index [0-4].
LabuteASA	The Labute ASA value for a molecule. A binned form of either the partial charge with a VSA. A binned form of either MolMR A binned form of either MolLogP.

data set [6]. This algorithm only considers the selected subset of features when splitting nodes [7].

- K-nearest neighbors (KNN): categorizes compounds based on the training instances that are nearest to them in feature space [8]. A compound is assigned to the class that is most popular among its q closest neighbors. The distance between data compounds is measured by Minkowski distance, and the value of q is set to three.
- Support Vector Machine (SVM): identifies the hyperplane dividing the various classes and maps the decision limit for each class [9]. This approach is based on a kernel function, which is a mapping operation done on the training set to increase its similarity to a set of data that can be separated. The non-linear Radial Basis Function (RBF) kernel is used in this implementation. It is uncommon to see linearly separable data in real-world situations; hence, the RBF kernel outperforms the logistic regression algorithm in terms of finding patterns [10].
- Gaussian Naive Bayes (GM): applies Bayes' theorem using the assumption of independence between each pair of features [11] and computes the probability that a given compound belongs to a given class. It belongs to a family of basic probabilistic classification techniques.

A total of 15 TMC' models (five ML algorithms and three molecular representations) were computed for each target without any feature filtering or selection with the available compound-target interaction data. The 30% of the total data was used for TCM evaluation (in addition to the VSD2 external validation data).

The applicability domain (AD) of each model was also computed. The AD refers to the region in space where the "normal" objects (compounds) were located [12], and it aims to address this by illustrating the chemical region outside of which forecasts cannot be regarded as confident [12, 13]. The AD was defined using a distance-based method (see Figure SM1.2) using the hamming distance for FGP descriptors and the euclidean distance for DSC descriptors. Additionally, the euclidean and hamming distances were applied simultaneously for evaluating AD using the FUS descriptors. For this procedure, the center of the training set was calculated using the standardized descriptors, and the maximum distance between the center and the training set compounds was taken as a maximum threshold [14]. If the distance between a query compound and the center was larger than this threshold, the query compound was considered outside the AD.

The AD perspective was established and validated for each compound in the training, and then it was used to evaluate the compounds of VSD2 before address the TCM models. Then, the confusion matrix was computed to evaluate the performance measurement for the TCM models. A confusion matrix, which is a very popular measurement, is a matrix that displays how well a classification model performs on a set of test data [15]. The confusion matrix depicts the percentages of the four classification outcomes that could be True Positive (TP), False Positive (FP), True Negative (TN), and False Negative (FN). TP are the interactions truly interact, TN are interactions that truly do not interact, FP are the interactions that are truly not interact,

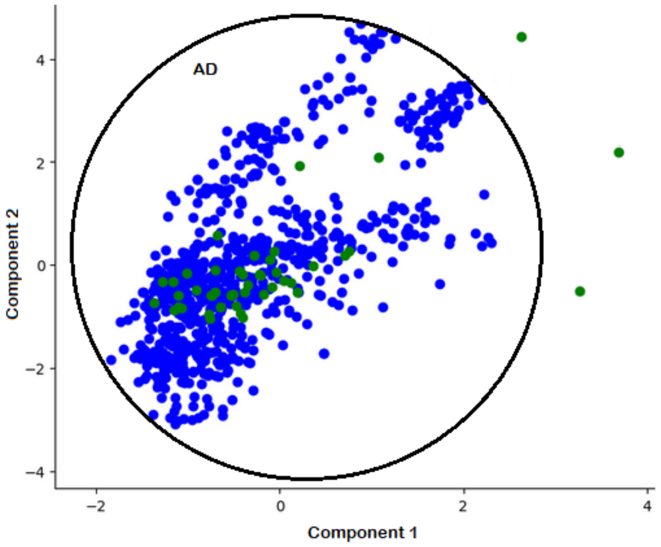

Fig. SM1.2 An example of a distance-based' applicability domain (AD) computed over the training sets of compounds (orange triangles) before training the target-centric model (TCM). AD' analysis helps to understand if the TCM can be used for any set of compounds. According to it, the testing sets of compounds (green dots) are assessed over the predefined AD. This means that, for a trustworthy prediction, compounds outside of AD are not considered.

but based on the ChEMBL[2] information, they were falsely denoted as positive interaction. and FN are the interactions that are truly interact, but based on the ChEMBL information, they were falsely denoted as negative interaction. These metrics are used to calculate other metrics, such as f1-score, that offer more insightful analyses of the prediction.

The results of the TCM evaluation in this section were reported in terms of the f1-score. The f1-score at its highest is 1, indicating perfect precision and recall; at its lowest possible value is 0.

Note SM1.3. Target-centric Models from Web Tools (WTCM

A collection of 17 publicly accessible WTCM models (Table SM1.2) that might be used as web tools are used for benchmarking. These models were taken from five websites [16] [17] [18] [19].

In [20] and [16], the *MolTarPred's web tool* publish a MTP model that uses similarity technique to identify the target profile of 10 size for small organic compounds. Similarity calculations o each compound are computed using the Morgan fingerprint. It takes into account a reliability score for the target profile which leads to higher prospective hit rates and where values less than three indicate a negative action.

[2]ChEMBL (https://www.ebi.ac.uk/chembl/)is a database maintained by the *European Bioinformatics Institute* collects data from scientific literature and contains compounds bioactivity data against targets

In [17], the *SwissTargetPrediction's web tool* publish the STP model that predicts around 100 protein targets for a small molecules based on a similarity principle though reverse screening. It uses a probability score for rating the target profile which encompasses also several species.

In [18], the *TargetNet's web tool* publish six models based on different compound descriptors: ECFP6 (TS-ECFP6), FP2 (TS-FP2), Daylight (TS-Dl), MACCCs (TS-MACCCs), ECFP2 (TS-ECFP2) and ECFP4 (TS-ECFP4).The prediction models were constructed using naïve bayes algorithm and a variety of molecular descriptors. To enhance the prediction capacity, ensemble learning from these fingerprints was also applied to show a total of 623 human targets in its target profile.

In [21], *Sea Bkslab web tool* publish the SB model that used a similarity method and a statistical model was developed to rank the significance. They also confirmed their predictions experimentally and report p-value score in the 30 size target profile. The scores values with a p-value under 0.05 represents an active interaction.

In [19], the *PPB2's web tool* publish eight models: Extended Connectivity fingerprint NN (ECfp4-NN), Shape and Pharmacophore fingerprint NN (Xfp-NN), Molecular Quantum Numbers (MQN-NN), Extended Connectivity fingerprint NN+NB (ECfp4-NN-NB), Shape and Pharmacophore fingerprint Xfp NN + ECfp4 NB (Xfp-NN-ECfp4-NB), Molecular Quantum Numbers MQN NN + ECfp4 NB (Xfp-NN-ECfp4-NB), Extended Connectivity fingerprint ECfp4 NB (ECFp4-NB), Extended Connectivity fingerprint DNN (ECFp4-DNN). Models exploited six different fingerprints and four fusion fingerprints using nearest neighbor (NN),naive bayes (NB) andd Deep Neural Network (DNN) machine learning algorithms to predict a 10 size target profile.

Compounds in VSD3 were used as input to WTCM algorithms using scraping strategies (Table SM1.2). Then, to evaluate the performance of these algorithms, the confusion matrix was also computed to determine the metrics of true positive rate (TPR), false positive rate (FPR), true negative rate (TNR), and false negative rate (FNR). TPR estimates the fraction of positive interactions that were identified as positive interactions. TNR estimates the fraction of negative interactions that were identified as negative interactions. FPR estimates the fraction of negative interactions identified as positive interactions. FNR estimates the fraction of positive interactions identified as negative interactions. These metrics were used to compared the WTCM with the models trained in the previous section (15 TCM).

Additionally, the recovery rate and unknown rate metrics were defined for each compound in VSD3. The recovery rate for a compound measured the fraction of all targets present in the ChEMBL database for which a prediction can be made by a specific method. That is, no prediction could be performed for a target if no model has been obtained for it. If a compound-target predicted association waas outside a model's AD, this prediction was excluded for computing the recovery rate. This metric was defined by Equation 1, where T_{ChEMBL} represents the number of targets present in the ChEMBL database. For a particular method (compound representation + ML algorithm), the overall recovery rate was computed as the mean of the metric across all compounds.

Table SM1.2 Target-centric models from web tools (WTCM).

Algorithms	Acronym	Last Update	Method	Score value	Order	Targets [6]
MolTarPred [16, 20]	MTP	6/19/2017	Similarity	Reliability[1]	Descending	10
Swiss Target Prediction[4] [17, 22]	STP	5/20/2019	Similarity	Probability[2]	Ascending	100
Targetnet Scbdd ECFP6 [18]	TS-ECFP6	5/11/2016	QSAR	Probability[2]	Ascending	623
Targetnet Scbdd FP2 [18]	TS-FP2	5/11/2016	QSAR	Probability[2]	Ascending	623
Targetnet Scbdd Daylight [18]	TS-Dl	5/11/2016	QSAR	Probability[2]	Ascending	623
Targetnet Scbdd MACCs [18]	TS-MACCs	5/11/2016	QSAR	Probability[2]	Ascending	623
Targetnet Scbdd ECFP2 [18]	TS-ECFP2	5/11/2016	QSAR	Probability[2]	Ascending	623
Targetnet Scbdd ECFP4 [18]	TS-ECFP4	5/11/2016	QSAR	Probability[2]	Ascending	623
Sea Bkslab [21]	SB	2/7/2017	Similarity	P-value[3]	Descending	30
Extended connectivity fingerprint NN [19]	ECfp4-NN	12/17/2018	Similarity	Order[5]	Ascending	20
Shape and pharmacophore fingerprint NN [19]	Xfp-NN	12/17/2018	Similarity	Order[5]	Ascending	20
Molecular quantum Numbers [19]	MQN-NN	12/17/2018	Similarity	Order[5]	Ascending	20
Extended connectivity fingerprint NN+NB [19]	ECfp4-NN-NB	12/17/2018	QSAR	Order[5]	Ascending	20
Shape and pharmacophore fingerprint Xfp NN+ECfp4 NB [19]	Xfp-NN-ECfp4-NB	12/17/2018	QSAR	Order[5]	Ascending	20
Molecular quantum numbers MQN NN+ECfp4 NB [19]	MQN-NN-ECfp4-NB	12/17/2018	QSAR	Order[5]	Ascending	20
Extended connectivity fingerprint ECfp4 NB [19]	ECFp4-NB	12/17/2018	QSAR	Order[5]	Ascending	20
Extended connectivity fingerprint DNN [19]	ECFp4-DNN	12/17/2018	QSAR	Order[5]	Ascending	20

Note: The target predictions of each web-tool were collected with scraping techniques.[1] The scores values with values under 3 represents negative activity. [2] The scores values with probability 0 represents an inactive interaction. [3] The scores values with a p-value under 0.05 represents an active interaction. [4] It only allows molecule smiles under 200 characters. [5] It does not provide inactive interactions, only the targets with potential interaction. [6] The number of predicted targets.

$$recovery = \frac{TP + FP + TN + FN}{T_{ChEMBL}} \tag{1}$$

On the other hand, the unknown rate represented the proportion of interactions predicted by a method (compound representation + ML algorithm) for a given compound that has no experimental information in ChEMBL to be assessed. This metric was evaluated across all targets for which a valid model (TCM) is available. As for the recovery rate, a prediction outside a model's AD was labeled as unknown. So, the difference between the number of targets in VSD3 (T_{VSD3}), and the total predicted targets inside the AD of the TCM (the sum of TP, FP, TN, and FP) was divided by the number of targets in VSD3 to obtain the unknown rate for each compound (Equation 2). For a particular method, the unknown rate was defined as the average of the metric across all compounds.

$$unknown = \frac{T_{VSD3} + (TP + FP + TN + FN)}{T_{VSD3}} \tag{2}$$

Note SM1.4. Consensus strategies

Each target-centric model from a web tool(WTCM) (Table SM1.2) had a particular way of ranking the compound' target profile, and its targets prediction scores had different scales. Hence, to integrate all their algorithm's predictions in a consensus approach, some transformations were made to have all the scores on the same scale. First, each output from a web-centric algorithm that represents the possible interaction between the compound j and the protein k $x_{j,k}$ was normalized considering the following equations based on their output type: 1) Equation 3 was used for algorithms that use probability scores [0-1], 2) Equation 4 was used for algorithms that use order scores [1-n], 3) Equation 5 was used for algorithms that use the reliability of prediction scores [1-n], and 4) Equation 6 was used for models using the p-value score [0-1] with a cutoff of 0.05.

$$y_{j,k} = \frac{x_{j,k} - min(x)}{max(x) - min(x)} \tag{3}$$

$$y_{j,k} = 1 - \frac{x_{j,k} - min(x)}{max(x) - 1} \tag{4}$$

$$y_{j,k} = \begin{cases} 0, & \text{if } x_{j,k} < cuttoff. \\ 1 - \frac{max(x) - x_{j,k}}{max(x) - cuttoff}, & \text{otherwise.} \end{cases} \tag{5}$$

$$y_{j,k} = \begin{cases} 0, & \text{if } x_{j,k} \geqslant 0.05. \\ 1 - \frac{0.05 - x_{j,k}}{0.05 - min(x)}, & \text{otherwise.} \end{cases} \tag{6}$$

Then, the consensus score ($z_{j,k}$) for the interaction between compound j and protein k across all algorithms published in the web tools i was computed as in eq.7.

$$z_{j,k} = \sqrt{< y_i^{j,k} > \frac{s_a}{s_t}} \tag{7}$$

In equation7, $< y_i^{j,k} >$ was the average of the normalized values obtained from equations 3, 4, 5, and 6 for a given compound-protein, s_a referred to the number of web-tools identifying the protein k and S_t was the total number of web tools. The consensus score z represented the reliability of the interaction between a compound under study and a particular target, considering the information across different algorithms and ranges from 0 to 1.

The consensus strategy over WTCM included all the algorithms that have information depending on the availability of the web tool. The predicted target list size (10 to 623 predictions, as shown in Table SM1.2) was also different for each algorithm, as mentioned previously. The consensus score was a ranking criterion for which a threshold of 0.5 was proposed to classify predictions of active or negative interactions. Several top-ranked fractions from 1% to 100% of the ranked list (step size of 5%) were analyzed to evaluate the performance of each fraction of the ranked lists. For each fraction of the ranked list, the consensus scores over 0.5 were considered positive interactions, while the remaining values were considered as negative ones.

Next, the predicted target profiles, over the consensus strategy with WTCM, were sorted descending by consensus score. The confusion matrix and the performance metrics [23] of TPR, TNR, FPR and FNR were calculated in each fraction of the ranked splits. Additionally, the metrics of recovery rate and unknown rate were computed as defined in the previous section. According to this viewpoint, the values that were at the top of the splits had the most accurate forecasts (it was some sort of initial enrichment usually applied in virtual screening).

In the case of trained target-centric models(TCM), the predicted target lists per compound were the same size regardless of the employed modeling method since there were the same trained models. Therefore, an analysis was done to keep a representation of the 15 models and to maintain their diversity (this process was not required with the previous WTCM models because the output target list is different for each algorithm) before performing the consensus strategy. Diversity in base predictors was one of the most important aspects to consider for fusion strategy methods since this avoids biases toward over-represented decision-makers.

The rand index (RI) metric was used to cluster the prediction's binary vectors according to their similarity. Output vectors for the VSD2 dataset were selected for clustering in the TCM. RI is one of the most known indices for measuring the similarity between two clusters and has been widely used in various studies [24]. It was used to create a similarity matrix to group TCM with hierarchical clustering. The RI is bound between 0 and 1, with 0 indicating that the binary vectors do not agree on any pair of points and 1 indicating that the data clustering is the same. The hierarchical clustering was built using the matrix of pairwise RI values over the 15 TCM, and three cutoffs were set to identify three different TCM groups. Three groups of TCM were generated, one per clustering threshold. The performance of the TCM groups was evaluated as described above for the fusion of WTCM.

$$z_{j,k} = \frac{\sum_{i=1}^{n} p_{k,i}}{n} \tag{8}$$

Then, as well as WTCM consensus, the consensus strategy was also performed over TCM for the group with the best performance over the compounds in VDS3.

The outputs of all TCM were membership class probabilities $p_{k,i}$ in the range [0, 1], so no normalization was required for their fusion. The mean across all probabilities of the sample models was computed (Equation 8), and the target profile was sorted descendingly using the differences in class probabilities (active probability minus inactive probability) as shown in. Then, different top-ranked subsets from 1% to 100% (step size of 5%) were also evaluated. The probability differences over 0 (equivalent to the 0.5 used in the algorithms of the web tools) were regarded as positive bioactivity, while the remaining values were regarded as negative bioactivity in each subset. Results were also reported in terms of the performance metrics of TPR, TNR, FPR, FNR, recovery rate, and unknown rate.

References

[1] Gaulton, A., Bellis, L.J., Bento, A.P., Chambers, J., Davies, M., Hersey, A., Light, Y., McGlinchey, S., Michalovich, D., Al-Lazikani, B., Overington, J.P.: ChEMBL: a large-scale bioactivity database for drug discovery. Nucleic Acids Research **40**(D1), 1100–1107 (2011) https://doi.org/10.1093/nar/gkr777 https://academic.oup.com/nar/article-pdf/40/D1/D1100/16955876/gkr777.pdf

[2] Papadatos, G., Davies, M., Dedman, N., Chambers, J., Gaulton, A., Siddle, J., Koks, R., Irvine, S.A., Pettersson, J., Goncharoff, N., Hersey, A., Overington, J.P.: Surechembl: a large-scale, chemically annotated patent document database. Nucleic Acids Research (D1), 1220–1228 (2015) https://doi.org/10.1093/nar/gkv1253 https://academic.oup.com/nar/article-pdf/44/D1/D1220/16661768/gkv1253.pdf

[3] Cereto-Massagué, A., Ojeda, M.J., Valls, C., Mulero, M., Garcia-Vallvé, S., Pujadas, G.: Molecular fingerprint similarity search in virtual screening. Methods **71**, 58–63 (2015) https://doi.org/10.1016/j.ymeth.2014.08.005 . Virtual Screening

[4] Landrum, G.: Rdkit: Open-source cheminformatics software (2016)

[5] Podgorelec, V., Zorman, M.: Decision trees. In: Encyclopedia of Complexity and Systems Science. Springer, ??? (2009). https://doi.org/10.1007/978-3-642-27737-5_117-2

[6] Belgiu, M., Drăguţ, L.: Random forest in remote sensing: A review of applications and future directions. ISPRS Journal of Photogrammetry and Remote Sensing **114**, 24–31 (2016) https://doi.org/10.1016/j.isprsjprs.2016.01.011

[7] Nguyen, T.-T., Huang, J.Z., Nguyen, T.T.: Unbiased feature selection in learning random forests for high-dimensional data. The Scientific World Journal **2015** (2015) https://doi.org/10.1155/2015/471371

[8] Guo, G., Wang, H., Bell, D., Bi, Y., Greer, K.: Knn model-based approach in classification, vol. 2888, pp. 986–996 (2003). https://doi.org/10.1007/

978-3-540-39964-3_62

[9] Awad, M., Khanna, R.: Support Vector Machines for Classification, pp. 39–66 (2015). https://doi.org/10.1007/978-1-4302-5990-9_3

[10] Ray, S.: A quick review of machine learning algorithms. In: 2019 International Conference on Machine Learning, Big Data, Cloud and Parallel Computing (COMITCon), pp. 35–39 (2019). https://doi.org/10.1109/COMITCon.2019.8862451

[11] Wickramasinghe, I., Kalutarage, H.: Naive bayes: applications, variations and vulnerabilities: a review of literature with code snippets for implementation indika. Soft Computing **24** (2021) https://doi.org/10.1007/s00500-020-05297-6

[12] Mathea, M., Klingspohn, W., Baumann, K.: Chemoinformatic classification methods and their applicability domain. Molecular Informatics **35** (2016) https://doi.org/10.1002/minf.201501019

[13] Sheridan, R.: Three useful dimensions for domain applicability in qsar models using random forest. Journal of chemical information and modeling **52**, 814–23 (2012) https://doi.org/10.1021/ci300004n

[14] Wang, Z., Chen, J., Hong, H.: Applicability domains enhance application of ppary agonist classifiers trained by drug-like compounds to environmental chemicals. Chemical Research in Toxicology **33**, 1382–1388 (2020) https://doi.org/10.1021/acs.chemrestox.9b00498

[15] Canbek, G., Sagiroglu, S., Temizel, T.T., Baykal, N.: Binary classification performance measures/metrics: A comprehensive visualized roadmap to gain new insights, 821–826 (2017) https://doi.org/10.1109/UBMK.2017.8093539

[16] Peón, A., Li, H., Ghislat, G., Leung, K., Wong, M.H., Lu, G., Ballester, P.: Moltarpred: A web tool for comprehensive target prediction with reliability estimation. Chemical Biology amd Drug Design **94**, 1390–1401 (2019) https://doi.org/10.1111/cbdd.13516

[17] Daina, A., Michielin, O., Zoete, V.: Swisstargetprediction: updated data and new features for efficient prediction of protein targets of small molecules. Nucleic Acids Research **47**(W1), 357–364 (2019) https://doi.org/10.1093/nar/gkz382 https://academic.oup.com/nar/article-pdf/47/W1/W357/28880175/gkz382.pdf

[18] Yao, Z., Dong, J., Che, Y.-J., Zhu, M.-F., Wen, M., Wang, N., Wang, S., Lu, A., Cao, D.-S.: Targetnet: a web service for predicting potential drug–target interaction profiling via multi-target sar models. Journal of Computer-Aided Molecular Design **30** (2016) https://doi.org/10.1007/s10822-016-9915-2

[19] Awale, M., Reymond, J.-L.: The polypharmacology browser ppb2: Target prediction combining nearest neighbors with machine learning. Journal of Chemical Information and Modeling **59** (2018) https://doi.org/10.1021/acs.jcim.8b00524

[20] Peón, A., Naulaerts, S., Ballester, P.: Predicting the reliability of drug-target interaction predictions with maximum coverage of target space. Scientific Reports **7** (2017) https://doi.org/10.1038/s41598-017-04264-w

[21] Keiser, M., Roth, B., Armbruster, B., Ernsberger, P., Irwin, J., Shoichet, B.: Relating protein pharmacology by ligand chemistry. Nature biotechnology **25**, 197–206 (2007) https://doi.org/10.1038/nbt1284

[22] Gfeller, D., Michielin, O., Zoete, V.: Shaping the interaction landscape of bioactive molecules. Bioinformatics **29**(23), 3073–3079 (2013) https://doi.org/10.1093/bioinformatics/btt540 https://academic.oup.com/bioinformatics/article-pdf/29/23/3073/48895534/bioinformatics_29_23_3073.pdf

[23] Tharwat, A.: Classification assessment methods: a detailed tutorial (2018) https://doi.org/10.1016/j.aci.2018.08.003

[24] Al-amri, R., Murugesan, R.K., Almutairi, M., Munir, K., Alkawsi, G., Baashar, Y.: A clustering algorithm for evolving data streams using temporal spatial hyper cube. Applied Sciences **12**(13) (2022) https://doi.org/10.3390/app12136523
