# Supplementary material for: Prediction of compound-target interaction using several artificial intelligence algorithms and comparison with a consensus-based strategy
Source: J Cheminform. 2024 Mar 7;16:27. doi: 10.1186/s13321-024-00816-1 (PMC10919000; doi:10.1186/s13321-024-00816-1)
Supplement: Supplementary file 2 — Additional file 2. Extended results. TCM. Comparison between TCM and WTCM. TCM. Consensus approach. Web-tool implementation. [file 13321_2024_816_MOESM2_ESM.pdf]

Supplementary Material 2

SM2. Extended Results

I. Target-centric Models (TCM)

Outcome SM2.1. Training Target-centric Models

The target-centric models (TCM) trained in this manuscript are available in the *github repository* at https://github.com/kbjimenes/target-identification. The average results obtained by the 253 models across the 30% of the data during the traning (dataset DS1) are presented in Table SM2.1 (see more detail about methodology in Supplementary Material 1, Note SM1.2).

Table SM2.1: Average performance measures of the target-centric models (TCM) across the 30% on DS1.

Model	Precision	Recall	F1-score	Specificity	Accuracy
			FGP		
FGP_DT	0.88 ± 0.11	0.78 ± 0.11	0.82 ± 0.09	0.89 ± 0.1	0.84 ± 0.08
FGP_GM	0.81 ± 0.12	0.87 ± 0.12	0.83 ± 0.09	0.79 ± 0.13	0.83 ± 0.84
FGP_KNN	0.92 ± 0.09	0.78 ± 0.15	0.82 ± 0.11	0.92 ± 0.09	0.84 ± 0.08
FGP_RF	0.91 ± 0.09	0.86 ± 0.11	0.88 ± 0.08	0.91 ± 0.09	0.89 ± 0.07
FGP_SVM	0.79 ± 0.18	0.67 ± 0.32	0.71 ± 0.18	0.77 ± 0.29	0.69 ± 0.16
			DSC		
DSC_DT	0.89 ± 0.08	0.82 ± 0.09	0.85 ± 0.07	0.89 ± 0.09	0.86 ± 0.06
DSC_GM	0.77 ± 0.16	0.63 ± 0.26	0.65 ± 0.12	0.76 ± 0.25	0.69 ± 0.12
DSC_KNN	0.91 ± 0.08	0.86 ± 0.09	0.88 ± 0.07	0.91 ± 0.08	0.88 ± 0.07
DSC_RF	0.91 ± 0.09	0.86 ± 0.08	0.88 ± 0.07	0.91 ± 0.09	0.88 ± 0.07
DSC_SVM	0.84 ± 0.1	0.81 ± 0.12	0.82 ± 0.08	0.84 ± 0.11	0.82 ± 0.08
			FUS		
FUS_DT	0.88 ± 0.1	0.79 ± 0.12	0.83 ± 0.09	0.89 ± 0.1	0.84 ± 0.08
FUS_GM	0.82 ± 0.11	0.85 ± 0.12	0.83 ± 0.08	0.82 ± 0.13	0.83 ± 0.08
FUS_KNN	0.91 ± 0.11	0.74 ± 0.17	0.8 ± 0.13	0.91 ± 0.12	0.83 ± 0.09
FUS_RF	0.92 ± 0.08	0.85 ± 0.09	0.88 ± 0.07	0.92 ± 0.07	0.88 ± 0.06
FUS_SVM	0.89 ± 0.09	0.88 ± 0.09	0.87 ± 0.07	0.89 ± 0.09	0.88 ± 0.07

TCM trained with the algorithms: Decision Tree (DT), Gaussian Naive Bayes (GM), K-nearest neighbors (KNN), Random Forest (RF), and Support Vector Machine (SVM) considering three groups of descriptors: a) Morgan's fingerprint (FGP), b) general physiochemical, structural, and topological molecular properties (DSC), and c) the fusion of both descriptors (FUS).

Table SM2.2: Average performance measures of the target-centric models (TCM) across the 30% on DS1 considering different splits of interactions.

Models	Total	10-20		20-40		40-60		60-80		80-100		More than 100	
		TCMs	f1-score	TCMs	f1-score	TCMs	f1-score	TCMs	f1-score	TCMs	f1-score	TCMs	f1-score
FGP													
FGP_DT	253	3	0.8 ± 0.2	38	0.8 ± 0.1	32	0.8 ± 0.1	18	0.8 ± 0.1	20	0.8 ± 0.1	142	0.8 ± 0.1
FGP_GM	253	3	0.9 ± 0.1	38	0.9 ± 0.1	32	0.9 ± 0.1	18	0.8 ± 0.1	20	0.9 ± 0.1	142	0.8 ± 0.1
FGP_KNN	253	3	0.9 ± 0.1	38	0.8 ± 0.2	32	0.8 ± 0.1	18	0.8 ± 0.2	20	0.8 ± 0.1	142	0.9 ± 0.1
FGP_RF	253	3	0.9 ± 0.1	38	0.8 ± 0.2	32	0.9 ± 0.1	18	0.9 ± 0.1	20	0.9 ± 0.1	142	0.9 ± 0.1
FGP_SVM	253	3	0.5 ± 0.1	38	0.6 ± 0.2	32	0.7 ± 0.2	18	0.6 ± 0.3	20	0.7 ± 0.2	142	0.7 ± 0.2
DSC													
DSC_DT	253	1	1	40	0.8 ± 0.1	29	0.8 ± 0.1	20	0.8 ± 0.1	18	0.8 ± 0.1	145	0.9
DSC_GM	253	1	0.8	40	0.8 ± 0.2	29	0.7 ± 0.2	20	0.7 ± 0.1	18	0.7 ± 0.2	145	0.6 ± 0.2
DSC_KNN	253	1	0.7	40	0.9 ± 0.1	29	0.9 ± 0.1	20	0.9 ± 0.1	18	0.9 ± 0.1	145	0.9
DSC_RF	253	1	0.8	40	0.9 ± 0.1	29	0.9 ± 0.1	20	0.8 ± 0.1	18	0.9 ± 0.1	145	0.9
DSC_SVM	253	1	0.9	40	0.8 ± 0.1	29	0.8 ± 0.1	20	0.8 ± 0.1	18	0.8 ± 0.1	145	0.8 ± 0.1
FUS													
FUS_DT	253	4	0.9 ± 0.1	41	0.8 ± 0.1	29	0.8 ± 0.1	20	0.8 ± 0.1	19	0.8 ± 0.1	140	0.8 ± 0.1
FUS_GM	253	4	0.8 ± 0.2	41	0.8 ± 0.1	29	0.9 ± 0.1	20	0.9 ± 0.1	19	0.9 ± 0.1	140	0.8 ± 0.1
FUS_KNN	253	4	0.8 ± 0.1	41	0.7 ± 0.2	29	0.7 ± 0.2	20	0.8 ± 0.1	19	0.8 ± 0.1	140	0.8 ± 0.1
FUS_RF	253	4	0.8 ± 0.1	41	0.9 ± 0.1	29	0.9 ± 0.1	20	0.9 ± 0.1	19	0.9	140	0.9
FU_SVM	253	4	0.9 ± 0.1	41	0.9 ± 0.1	29	0.9 ± 0.1	20	0.9 ± 0.1	19	0.9 ± 0.1	140	0.9

TCM trained with the algorithms: Decision Tree (DT), Gaussian Naive Bayes (GM), K-nearest neighbors (KNN), Random Forest (RF), and Support Vector Machine (SVM) considering three groups of descriptors: a) Morgan's fingerprint (FGP), b) general physiochemical, structural, and topological molecular properties (DSC), and c) the fusion of both descriptors (FUS). Results evidence the f1-score achieved by the TCMs across different size of compound-target interactions.

Additionally, during the training testing over DS1, some splits were done over the 253 models due to observe the training behavior across the size of compound-target interactions. Table SM2.2 shows that most of the TCMs have more than 20 positive and 20 negative interactions. Similar results were achieve with 10 and 100 positive/negative interactions.

The results of the external validation with VDS2 and presented in Figure 1 of the main manuscript are fully presented in Table SM2.3

Table SM2.3: Average performance measures of the target-centric models (TCM) across the external validation dataset on VDS2.

Model	Precision	Recall	F1-score	Specificity	Accuracy
			FGP		
FGP_DT	0.76 ± 0.21	0.59 ± 0.21	0.64 ± 0.18	0.47 ± 0.27	0.57 ± 0.14
FGP_GM	0.76 ± 0.21	0.81 ± 0.26	0.77 ± 0.19	0.26 ± 0.29	0.69 ± 0.19
FGP_KNN	0.81 ± 0.23	0.44 ± 0.29	0.54 ± 0.22	0.67 ± 0.34	0.5 ± 0.18
FGP_RF	0.79 ± 0.21	0.69 ± 0.29	0.71 ± 0.23	0.47 ± 0.33	0.67 ± 0.19
FGP_SVM	0.82 ± 0.19	0.54 ± 0.39	0.66 ± 0.27	0.58 ± 0.41	0.57 ± 0.25
			DSC		
DSC_DT	0.76 ± 0.22	0.62 ± 0.23	0.67 ± 0.2	0.47 ± 0.27	0.61 ± 0.16
DSC_GM	0.78 ± 0.22	0.58 ± 0.29	0.61 ± 0.26	0.54 ± 0.32	0.57 ± 0.19
DSC_KNN	0.78 ± 0.21	0.69 ± 0.21	0.71 ± 0.18	0.46 ± 0.26	0.65 ± 0.15
DSC_RF	0.79 ± 0.21	0.69 ± 0.25	0.72 ± 0.21	0.47 ± 0.29	0.67 ± 0.18
DSC_SVM	0.78 ± 0.22	0.66 ± 0.28	0.69 ± 0.22	0.51 ± 0.31	0.64 ± 0.19
			FUS		
FUS_DT	0.76 ± 0.23	0.61 ± 0.22	0.67 ± 0.19	0.47 ± 0.27	0.59 ± 0.16
FUS_GM	0.76 ± 0.21	0.79 ± 0.27	0.76 ± 0.21	0.27 ± 0.31	0.69 ± 0.19
FUS_KNN	0.81 ± 0.22	0.46 ± 0.27	0.54 ± 0.23	0.68 ± 0.29	0.51 ± 0.19
FUS_RF	0.79 ± 0.21	0.73 ± 0.28	0.74 ± 0.23	0.45 ± 0.32	0.69 ± 0.19
FUS_SVM	0.77 ± 0.21	0.82 ± 0.29	0.8 ± 0.19	0.27 ± 0.31	0.72 ± 0.2

TCM trained with the algorithms: Decision Tree (DT), Gaussian Naive Bayes (GM), K-nearest neighbors (KNN), Random Forest (RF), and Support Vector Machine (SVM) considering three groups of descriptors: a) Morgan's fingerprint (FGP), b) general physiochemical, structural, and topological molecular properties (DSC), and c) the fusion of both descriptors (FUS).

Each target has an target space which was considered for training inside the application domain(AD). The AD (detailed in the methodology) refers to the average distance from the compounds to the centroid. Table SM2.4 indicates these threshold distance measures which gives us a reference of how similar are the compounds to be trained by each target. For modeling TCM, the tanimoto distance is used for evaluating the Morgan Fingerprint descriptors (FGP), the euclidean distance is used for molecular physiochemical descriptors (DSC) and both of them for the fusion of both descriptors (FUS). The threshold distance using the training and also average distance achieved by the compounds of the external validation dataset (VDS2) is presented.

Table SM2.4: Application domain threshold distance calculated across DS1 for FGP and DSC descriptors

			FGP		DSC	
No.	Target ID	Uniprot ID	Threshold	Average on VSD2	Threshold	Average on VSD2
1	3	O76074	0.21	0.1	425.24	9.01
2	4	O95180	0.17	0.11	147.96	12.23
3	6	P00374	0.18	0.08	710.02	10.72
4	8	P00519	0.3	0.09	676.79	12.33
5	9	P00533	0.31	0.12	2087.82	10.53
6	11	P00734	0.34	0.1	546.91	9.65
7	12	P00747	0.22	0.11	373.72	9.61
8	19	P03372	0.34	0.12	1033.43	14.23
9	23	P03952	0.46	0.12	202.74	10.59
10	38	P07099	0.13	0.05	276.91	12.62
11	47	P08172	0.24	0.08	163.37	9.25
12	55	P09917	0.19	0.1	1862.13	11.17
13	56	P10275	0.29	0.09	257.15	10.36
14	62	P11387	0.25	0.13	137.23	8.15
15	63	P11388	0.21	0.09	268.98	7.6
16	65	P11511	0.18	0.07	536.06	10.87
17	69	P12821	0.22	0.08	598.5	9.53
18	71	P14324	0.11	0.08	325.07	20.33
19	72	P14416	0.2	0.09	300.35	8.87
20	73	P14679	0.21	0.06	129.74	13.32
21	76	P15538	0.18	0.07	905.35	10.9
22	86	P21397	0.18	0.08	1909.22	11.32
23	93	P22303	0.27	0.11	4124.04	10.23
24	96	P23219	0.4	0.09	1546.69	10.5
25	100	P23975	0.33	0.09	342.88	14.17
26	104	P27338	0.47	0.09	1410	9.65
27	121	P31645	0.19	0.11	1265.24	13.97
28	126	P35354	0.4	0.09	642.32	10.27
29	129	P35372	0.3	0.14	907.08	15.59
30	133	P37231	0.19	0.11	770.72	10.53
31	155	Q01959	0.33	0.07	385.43	12.5
32	157	Q02127	0.13	0.09	677.78	14.19
33	163	Q07869	0.18	0.14	348.79	13.92
34	165	Q12809	0.28	0.1	6250.1	10.39
35	170	Q14432	0.16	0.09	270.28	9.97
36	174	Q92731	0.18	0.09	1500.28	34.95
37	194	P00742	0.22	0.12	2203.37	23.2
38	235	P08246	0.25	0.1	1406.57	14.39
39	242	P15121	0.17	0.07	349.51	10.59

Continued on next page...

Table SM2.4 – continued from previous page.

			FGP		DSC	
No.	Target ID	Uniprot ID	Threshold	Average on VSD2	Threshold	Average on VSD2
40	252	P29274	0.27	0.1	189.76	11.73
41	10003	P07339	0.23	0.13	1692.18	12.82
42	10009	O43613	0.22	0.11	1686.42	9.89
43	10044	P68400	0.2	0.08	469.7	13.75
44	10056	P78527	0.27	0.1	854.48	9.11
45	10069	P49862	0.2	0.1	48.14	12.12
46	10108	O95749	0.11	0.07	134.75	17.61
47	10140	P06239	0.31	0.11	1488.29	9.92
48	10141	P00338	0.19	0.13	243.51	16.11
49	10163	P10632	0.4	0.11	337.91	13.82
50	10179	Q03405	0.27	0.13	28.68	15.81
51	10188	Q16539	0.24	0.11	589.76	10.32
52	10189	P15090	0.11	0.09	69.43	12.45
53	10193	P00915	0.15	0.08	67.36	8.74
54	10197	P49841	0.35	0.12	526.99	11.67
55	10200	P14174	0.15	0.07	310.28	8.85
56	10216	Q05513	0.15	0.09	164.78	8.33
57	10258	P17706	0.23	0.12	485.01	11.3
58	10260	Q8NER1	0.17	0.09	2476.22	11.37
59	10266	P11362	0.17	0.12	1060.89	10.37
60	10278	Q04760	0.15	0.07	75.35	9.38
61	10280	Q9Y5N1	0.14	0.09	521.13	11.11
62	10323	Q96IY4	0.21	0.08	178.83	29.54
63	10332	P30304	0.17	0.05	145.84	9.15
64	10368	P48730	0.18	0.1	231.24	12.05
65	10378	P07858	0.22	0.09	713.34	10.39
66	10434	P12931	0.31	0.12	2732.09	8.67
67	10473	P61073	0.5	0.15	676.21	10.77
68	10495	P43235	0.22	0.09	1272.31	12.17
69	10498	P07711	0.18	0.11	71.61	16.26
70	10531	O96017	0.19	0.13	527.17	13.21
71	10532	P06276	0.22	0.11	718.89	10.07
72	10594	O00408	0.16	0.1	786.1	10.64
73	10599	Q07343	0.35	0.09	1315.93	10.86
74	10635	P36544	0.4	0.12	144.39	12.5
75	10656	P05067	0.27	0.1	159.91	8.99
76	10695	P31751	0.19	0.11	464.32	10.91
77	10711	P25098	0.17	0.1	336.04	10.79
78	10733	P04062	0.15	0.1	195.55	13.63
79	10773	P25025	0.14	0.08	692.09	9.78

Continued on next page...

Table SM2.4 – continued from previous page.

No.	Target ID	Uniprot ID	FGP Threshold	FGP Average on VSD2	DSC Threshold	DSC Average on VSD2
80	10781	Q96GD4	0.21	0.11	1474.47	10.92
81	10849	P52333	0.23	0.1	1327.44	10.54
82	10869	Q9BY41	0.36	0.1	1338.17	10.7
83	10899	Q15119	0.17	0.09	641.65	19.36
84	10901	P41743	0.14	0.08	383.12	15.31
85	10907	P53350	0.32	0.11	741.42	8.53
86	10919	P23458	0.23	0.1	1607.55	11.48
87	10938	O60674	0.25	0.1	2262.79	10.36
88	10945	Q969S8	0.23	0.1	260.88	10.18
89	10980	P35968	0.34	0.11	7470.07	11.86
90	11017	P49840	0.24	0.09	424.7	9
91	11024	P45452	0.23	0.09	2203.25	13.35
92	11109	P08254	0.23	0.11	1307.26	15.31
93	11110	P22894	0.23	0.09	655.75	11.48
94	11130	P00352	0.12	0.09	104.27	17.67
95	11140	P27487	0.22	0.1	872.46	10.95
96	11177	O00329	0.2	0.11	1060.43	11.13
97	11180	Q96RI1	0.35	0.11	242.86	8.44
98	11206	Q92769	0.32	0.12	1204.25	11.4
99	11208	O15379	0.35	0.12	1285.32	12.71
100	11213	P07900	0.26	0.1	417.54	11.61
101	11232	P56524	0.36	0.09	572.91	11.25
102	11242	Q05397	0.2	0.11	1161.57	11.98
103	11288	P00748	0.13	0.08	77.8	10.82
104	11291	O00519	0.19	0.11	1234.32	10.91
105	11307	Q9UBN7	0.36	0.1	2521.27	10.27
106	11356	P48775	0.13	0.08	113.03	13.18
107	11359	Q08499	0.18	0.1	391.19	10.14
108	11362	P42336	0.24	0.12	4171.1	12.77
109	11365	P10635	0.24	0.1	1154.46	10.98
110	11398	P08183	0.28	0.11	783.31	8.71
111	11400	P42345	0.29	0.12	1804.89	15.67
112	11415	P09467	0.12	0.06	264.76	15.86
113	11451	P08581	0.21	0.13	2824.17	10.35
114	11472	P40763	0.22	0.13	615.9	15.39
115	11480	Q14524	0.58	0.11	1190.78	7.02
116	11489	P28845	0.16	0.09	1536.88	12.86
117	11507	O00748	0.13	0.07	99.81	17.06
118	11520	P29350	0.19	0.11	172.24	9.54
119	11523	Q06124	0.2	0.1	252.13	12.64

Continued on next page...

Table SM2.4 – continued from previous page.

No.	Target ID	Uniprot ID	FGP Threshold	FGP Average on VSD2	DSC Threshold	DSC Average on VSD2
120	11565	P30305	0.2	0.08	489.57	9.98
121	11570	P17405	0.14	0.08	94.39	8.54
122	11574	P25024	0.19	0.09	237.25	11.34
123	11638	P28482	0.52	0.13	2823.26	9.01
124	11639	P27361	0.21	0.11	159.8	6.61
125	11663	P09874	0.17	0.11	1921.19	35.37
126	11678	P24941	0.19	0.11	1451.25	11.22
127	11723	P19971	0.14	0.04	149.33	9.95
128	11727	P34913	0.17	0.09	439.59	12.18
129	11797	Q9UKV0	0.22	0.09	153.48	10.34
130	11902	P04629	0.21	0.11	2489.92	12.38
131	11910	Q13946	0.16	0.1	603.88	11.85
132	11939	P48736	0.21	0.11	1918.6	10.98
133	11942	P00749	0.23	0.1	328.81	10.18
134	11968	P48147	0.4	0.12	338.85	13.84
135	12000	P08709	0.43	0.12	132.96	10.01
136	12021	Q96DB2	0.21	0.1	207.12	12.05
137	12030	P33527	0.26	0.09	282.8	7.67
138	12090	Q13627	0.17	0.07	334.94	11.08
139	12227	Q03181	0.16	0.14	343.45	34
140	12252	P56817	0.32	0.1	1542.16	9.76
141	12261	P45983	0.45	0.1	620.44	10.48
142	12283	Q9UQL6	0.17	0.09	149.73	11.22
143	12443	Q8WUI4	0.18	0.08	157.52	9.92
144	12576	P42338	0.21	0.11	545.94	11.28
145	12584	Q9Y337	0.23	0.1	50.17	11.57
146	12592	P14780	0.23	0.09	1838.14	11.18
147	12594	P05177	0.21	0.08	1008.66	9.91
148	12670	P36888	0.2	0.11	840.23	10.19
149	12694	P29597	0.23	0.11	889.55	11.93
150	12697	Q13547	0.36	0.11	4031.04	11.05
151	12704	P02766	0.12	0.07	62.93	10.9
152	12724	P51812	0.24	0.1	376.74	10.79
153	12855	P30419	0.17	0.09	311.99	18.87
154	12883	Q13526	0.39	0.1	184.64	6.54
155	12911	P11712	0.26	0.1	992.91	10.08
156	12912	P33261	0.26	0.11	1106.25	10.94
157	12920	P35610	0.26	0.11	411.35	11.86
158	12947	Q00535	0.15	0.1	161.31	12.69
159	13001	P08253	0.24	0.1	1394.93	11.26

Continued on next page...

Table SM2.4 – continued from previous page.

No.	Target ID	Uniprot ID	FGP Threshold	FGP Average on VSD2	DSC Threshold	DSC Average on VSD2
160	13005	P06870	0.17	0.12	96.9	17.95
161	13061	P18031	0.27	0.11	1249.73	10.1
162	17045	P08684	0.26	0.11	4093.13	10.1
163	17050	P08236	0.12	0.09	79.22	11.07
164	18036	P13866	0.18	0.12	393.69	13.56
165	19905	Q99705	0.18	0.12	2117.95	12.95
166	20014	O14965	0.25	0.11	2403.16	14.84
167	20073	P11509	0.11	0.03	273.29	9.64
168	20085	P20813	0.17	0.08	187.52	12.17
169	20131	Q00987	0.42	0.22	926.78	28.99
170	20132	Q01469	0.09	0	77.13	18.89
171	20137	Q13315	0.16	0.12	196.13	12.4
172	20139	Q13535	0.14	0.09	221.65	10.13
173	20143	Q16853	0.13	0.07	335.86	11.43
174	30017	P51955	0.16	0.09	56.11	12.05
175	30025	Q13153	0.28	0.1	140.2	9.09
176	100010	Q9Y233	0.22	0.09	3464.26	11.07
177	100079	P07949	0.17	0.11	526.44	12.97
178	100097	Q06187	0.3	0.13	2083.42	9.93
179	100100	O43497	0.15	0.12	111.93	14.66
180	100126	P15056	0.21	0.11	3557.26	9.76
181	100193	Q99685	0.14	0.09	474.99	12.93
182	100304	P10415	0.45	0.19	723.62	10.41
183	100325	Q16665	0.2	0.12	227.54	18.1
184	100410	P30530	0.2	0.13	470.55	16.46
185	100417	Q9UM73	0.25	0.13	1502.33	10.58
186	100446	Q9NTG7	0.31	0.18	201.9	26.12
187	100447	Q8IXJ6	0.33	0.12	773.36	12.95
188	100448	Q96EB6	0.3	0.1	564.24	12.18
189	100468	Q07820	0.41	0.13	668.1	21.16
190	100481	P42330	0.14	0.08	441.35	9.49
191	100485	O75365	0.13	0.07	72.1	26.55
192	100594	Q13822	0.19	0.12	457.33	9.41
193	100613	P49721	0.23	0.12	206.32	8.41
194	100624	P28074	0.23	0.12	669.49	10.63
195	100627	P24666	0.15	0.07	131.97	14.78
196	100633	P20618	0.23	0.11	187.62	8.92
197	100643	P14902	0.21	0.09	985.3	15.16
198	100781	O14786	0.36	0.18	68	17.07
199	100848	Q15391	0.23	0.11	61.31	14.74

Continued on next page...

Table SM2.4 – continued from previous page.

No.	Target ID	Uniprot ID	FGP Threshold	FGP Average on VSD2	DSC Threshold	DSC Average on VSD2
200	100874	Q09472	0.49	0.11	144.97	9.41
201	100878	Q99873	0.17	0.09	160.23	8.6
202	100912	Q06418	0.2	0.13	286.39	14.67
203	100933	Q9UGN5	0.14	0.11	182.49	13.58
204	100974	Q9UNQ0	0.26	0.1	343.87	13.5
205	101044	Q6P179	0.14	0.11	122.45	14.54
206	101181	Q86X55	0.16	0.08	128.23	8.72
207	101234	O00767	0.15	0.09	583.59	12.67
208	101310	Q92793	0.24	0.1	431.22	10.93
209	101351	P52895	0.13	0.08	201.38	11.9
210	101379	P22413	0.13	0.08	43.05	10.37
211	101412	O75762	0.14	0.09	224.86	11.65
212	101476	P27695	0.15	0	162.21	36.46
213	101589	P35222	0.39	0.15	46.78	8.59
214	101608	Q9NYK1	0.17	0.1	111.95	9.62
215	101611	Q9NZ08	0.15	0.1	93.93	19.26
216	102420	P34947	0.36	0.14	82.6	9.39
217	102439	O60341	0.46	0.1	632.16	9.25
218	102672	Q96KQ7	0.17	0.11	449.84	10.89
219	103061	Q12931	0.2	0.08	67.21	12.02
220	103069	Q9NUW8	0.19	0.12	235.04	12.9
221	103079	P55072	0.15	0.1	328.06	13.84
222	103165	P61964	0.39	0.1	94.83	10.48
223	103167	Q7Z2W7	0.13	0.09	417.17	12.18
224	103454	O60885	0.21	0.1	553.68	12.17
225	103481	P01106	0.09	0.07	51.4	13.16
226	103590	Q96LA8	0.14	0.08	173.71	14.4
227	103655	Q9Y239	0.19	0.12	74.27	11.97
228	103659	B2RXH2	0.15	0.07	67.52	9.24
229	103699	Q9HC29	0.19	0.12	95.41	9.13
230	103722	P25440	0.14	0.09	413.42	14.42
231	103726	P48145	0.16	0.09	230.24	14.25
232	103982	P51449	0.17	0.11	429.71	12.44
233	104004	Q96P20	0.15	0.08	107.38	12.69
234	104017	Q9Y4P1	0.21	0.07	333.34	9.96
235	104197	Q8TEK3	0.19	0.11	61.91	12.13
236	104265	Q58F21	0.14	0.09	89.88	11.41
237	104266	Q15059	0.14	0.1	378.43	14.14
238	104329	Q9P0U3	0.13	0.11	96.49	13.36
239	104483	O75874	0.19	0.12	1305.75	16.7

Continued on next page...

Table SM2.4 – continued from previous page.

No.	Target ID	Uniprot ID	FGP		DSC	
			Threshold	Average on VSD2	Threshold	Average on VSD2
240	104486	P54829	0.16	0.11	155.19	14.41
241	104568	Q5XXA6	0.14	0.09	121.7	17.87
242	105188	Q6PL18	0.12	0.1	88.35	24.56
243	105196	Q93009	0.16	0.11	128.17	11.38
244	105223	Q9NXA8	0.33	0.25	122.32	14.41
245	105247	O95551	0.14	0.1	416.83	12.25
246	105346	P00746	0.16	0.11	1113.61	17.55
247	105596	O43175	0.16	0.13	90.92	24.87
248	107664	Q53GL7	0.13	0.07	89	11.44
249	108027	Q8NHU3	0.16	0.09	96.01	13.33
250	109560	Q9NP59	0.11	0	188.59	29.96
251	109917	Q9UDY8	0.11	0.08	99.42	10.9
252	116091	P46095	0.14	0.11	63.73	11.74
253	117391	P41182	0.29	0.12	46.21	11.99

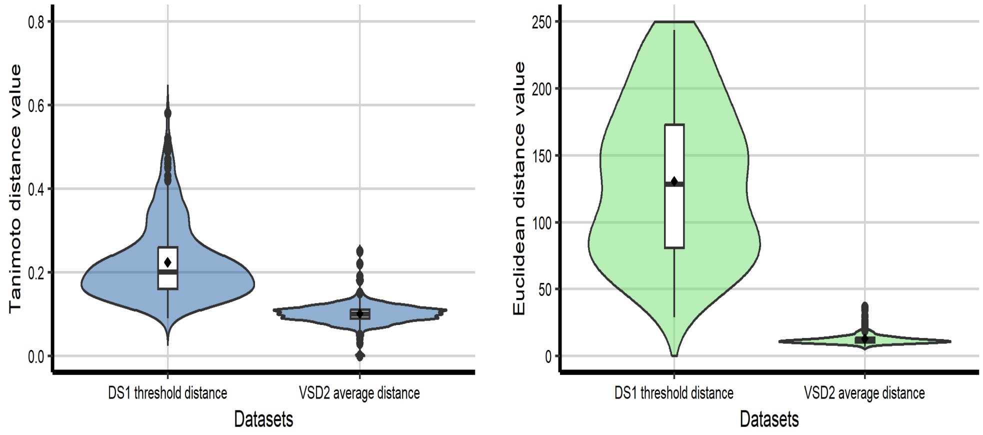

(a) FGP

(b) DSC

Fig. SM2.1: Distance threshold computed over DS1 and the average distance of compound-target interactions on VSD2 with Morgan Fingerprint (FGP) and physichochemical descriptors (DSC)

The graphical representation of these distances' distribution on the Table SM2.4 are in Fig SM2.1 for both group of descriptors FGP and DSC.It indicates that the distance is lower for the external data (VSD2). This reduction in the average distance

is a consequence of the process of validating compounds with the application domain (AD) imposed in the screening of the external data.

Additionally, a Tukey's Test was computed over the f1-scores achieved by the TCM on the external validation dataset on VDS2 due to find which groups show evidence of differing from one another. The purpose of the Turkey test is to identify means that are statistically distinct from one another by using a single-step multiple comparison technique [1]. Table SM2.5 displays the comparisons between every pair of models. The adjusted p-value identifies the pairs that are significantly difference. When compared values are less than the significance level, the difference between those group means is statistically significant. For example, the group FUS_RF & DSC_DT difference is statistically significant while using a family error rate of 0.05. The mean difference between these two groups is 0.068.

Table SM2.5: Results of Tukey's Test over target-centric models (TCM) across the external validation dataset on VDS2.

Pairs of models	Mean difference	Lower estimation	Upper estimation	Adjusted p-value
DSC_GM & DSC_DT	-0.058	-0.123	0.008	0.156
DSC_KNN & DSC_DT	0.046	-0.019	0.112	0.514
DSC_RF & DSC_DT	0.052	-0.014	0.117	0.325
DSC_SVM & DSC_DT	0.023	-0.043	0.088	0.998
FGP_DT & DSC_DT	-0.025	-0.09	0.041	0.995
FGP_GM & DSC_DT	0.101	0.035	0.167	0.0
FGP_KNN & DSC_DT	-0.131	-0.197	-0.064	0
FGP_RF & DSC_DT	0.0462	-0.019	0.112	0.535
FGP_SVM & DSC_DT	-0.009	-0.079	0.059	1
FUS_DT & DSC_DT	-0.004	-0.07	0.061	1
FUS_GM & DSC_DT	0.088	0.022	0.154	0.0
FUS_KNN & DSC_DT	-0.127	-0.193	-0.061	0
FUS_RF & DSC_DT	0.068	0.002	0.134	0.033
FUS_SVM & DSC_DT	0.131	0.064	0.197	0
DSC_KNN & DSC_GM	0.104	0.039	0.169	0.0
DSC_RF & DSC_GM	0.109	0.0437	0.175	0.0
DSC_SVM & DSC_GM	0.0803	0.014	0.146	0.0
FGP_DT & DSC_GM	0.0333	-0.032	0.099	0.928
FGP_GM & DSC_GM	0.159	0.093	0.225	0
FGP_KNN & DSC_GM	-0.073	-0.139	-0.006	0.017
FGP_RF & DSC_GM	0.104	0.038	0.17	0.0
FGP_SVM & DSC_GM	0.048	-0.021	0.117	0.552
FUS_DT & DSC_GM	0.053	-0.012	0.119	0.273
FUS_GM & DSC_GM	0.146	0.079	0.212	0
FUS_KNN & DSC_GM	-0.069	-0.136	-0.003	0.029
FUS_RF & DSC_GM	0.126	0.060	0.192	0
FUS_SVM & DSC_GM	0.189	0.122	0.255	0

Continued on next page...

Table SM2.5 – continued from previous page.

Pairs of models	Mean difference	Lower estimation	Upper estimation	Adjusted p-value
DSC_RF & DSC_KNN	0.005	-0.06	0.0708	1
DSC_SVM & DSC_KNN	-0.024	-0.089	0.042	0.997
FGP_DT & DSC_KNN	-0.071	-0.136	-0.005	0.021
FGP_GM & DSC_KNN	0.055	-0.011	0.121	0.225
FGP_KNN & DSC_KNN	-0.177	-0.243	-0.11	0
FGP_RF & DSC_KNN	-0.0	-0.066	0.066	1
FGP_SVM & DSC_KNN	-0.056	-0.125	0.013	0.27
FUS_DT & DSC_KNN	-0.051	-0.116	0.015	0.361
FUS_GM & DSC_KNN	0.042	-0.024	0.108	0.699
FUS_KNN & DSC_KNN	-0.173	-0.239	-0.107	0
FUS_RF & DSC_KNN	0.022	-0.041	0.088	0.998
FUS_SVM & DSC_KNN	0.0846	0.018	0.15	0.0
DSC_SVM & DSC_RF	-0.029	-0.095	0.037	0.977
FGP_DT & DSC_RF	-0.076	-0.142	-0.01	0.0
FGP_GM & DSC_RF	0.049	-0.016	0.116	0.395
FGP_KNN & DSC_RF	-0.182	-0.249	-0.116	0
FGP_RF & DSC_RF	-0.005	-0.071	0.061	1
FGP_SVM & DSC_RF	-0.061	-0.131	0.008	0.153
FUS_DT & DSC_RF	-0.056	-0.122	0.01	0.208
FUS_GM & DSC_RF	0.037	-0.029	0.102	0.866
FUS_KNN & DSC_RF	-0.179	-0.245	-0.112	0
FUS_RF & DSC_RF	0.017	-0.049	0.083	0.999
FUS_SVM & DSC_RF	0.079	0.013	0.146	0.005
FGP_DT & DSC_SVM	-0.047	-0.113	0.019	0.501
FGP_GM & DSC_SVM	0.079	0.0123	0.145	0.0
FGP_KNN & DSC_SVM	-0.153	-0.219	-0.086	0
FGP_RF & DSC_SVM	0.024	-0.043	0.089	0.997
FGP_SVM & DSC_SVM	-0.032	-0.102	0.037	0.963
FUS_DT & DSC_SVM	-0.0268	-0.093	0.039	0.989
FUS_GM & DSC_SVM	0.066	-0.0	0.132	0.056
FUS_KNN & DSC_SVM	-0.15	-0.216	-0.083	0
FUS_RF & DSC_SVM	0.046	-0.02	0.112	0.552
FUS_SVM & DSC_SVM	0.108	0.042	0.175	0.0
FGP_GM & FGP_DT	0.126	0.059	0.192	0
FGP_KNN & FGP_DT	-0.106	-0.173	-0.039	0.0
FGP_RF & FGP_DT	0.071	0.004	0.137	0.024
FGP_SVM & FGP_DT	0.015	-0.055	0.084	0.999
FUS_DT & FGP_DT	0.02	-0.046	0.086	0.999
FUS_GM & FGP_DT	0.112	0.046	0.179	0.0
FUS_KNN & FGP_DT	-0.103	-0.169	-0.036	0.0
FUS_RF & FGP_DT	0.093	0.027	0.159	0.0

Continued on next page...

Table SM2.5 – continued from previous page.

Pairs of models	Mean difference	Lower estimation	Upper estimation	Adjusted p-value
FUS_SVM & FGP_DT	0.155	0.088	0.222	0
FGP_KNN & FGP_GM	-0.232	-0.299	-0.165	0
FGP_RF & FGP_GM	-0.055	-0.122	0.011	0.238
FGP_SVM & FGP_GM	-0.111	-0.181	-0.042	0.0
FUS_DT & FGP_GM	-0.106	-0.172	-0.039	0.0
FUS_GM & FGP_GM	-0.014	-0.079	0.053	0.999
FUS_KNN & FGP_GM	-0.229	-0.295	-0.162	0
FUS_RF & FGP_GM	-0.0332	-0.099	0.033	0.936
FUS_SVM & FGP_GM	0.0293	-0.038	0.096	0.979
FGP_RF & FGP_KNN	0.177	0.109	0.244	0
FGP_SVM & FGP_KNN	0.121	0.051	0.191	0.0
FUS_DT & FGP_KNN	0.126	0.059	0.193	0
FUS_GM & FGP_KNN	0.219	0.152	0.286	0
FUS_KNN & FGP_KNN	0.004	-0.064	0.071	1
FUS_RF & FGP_KNN	0.199	0.132	0.266	0
FUS_SVM & FGP_KNN	0.261	0.194	0.329	0
FGP_SVM & FGP_RF	-0.056	-0.126	0.014	0.291
FUS_DT & FGP_RF	-0.051	-0.117	0.016	0.383
FUS_GM & FGP_RF	0.042	-0.025	0.108	0.712
FUS_KNN & FGP_RF	-0.173	-0.24	-0.108	0
FUS_RF & FGP_RF	0.022	-0.044	0.089	0.999
FUS_SVM & FGP_RF	0.085	0.017	0.152	0.002
FUS_DT & FGP_SVM	0.005	-0.064	0.075	1
FUS_GM & FGP_SVM	0.098	0.028	0.167	0.0
FUS_KNN & FGP_SVM	-0.117	-0.187	-0.048	0.0
FUS_RF & FGP_SVM	0.078	0.008	0.148	0.012
FUS_SVM & FGP_SVM	0.141	0.070	0.211	0
FUS_GM & FUS_DT	0.092	0.0261	0.159	0.0
FUS_KNN & FUS_DT	-0.123	-0.189	-0.056	0
FUS_RF & FUS_DT	0.073	0.006	0.139	0.0166
FUS_SVM & FUS_DT	0.135	0.0682	0.202	0
FUS_KNN & FUS_GM	-0.215	-0.282	-0.149	0
FUS_RF & FUS_GM	-0.0197	-0.086	0.047	0.999
FUS_SVM & FUS_GM	0.043	-0.024	0.109	0.687
FUS_RF & FUS_KNN	0.195	0.129	0.262	0
FUS_SVM & FUS_KNN	0.258	0.191	0.325	0
FUS_SVM & FUS_RF	0.063	-0.005	0.129	0.099

II. Target-centric models (TCM) with models from web tools (WTCM).

Outcome SM2.2. Scraping target-profile from WTCM predictions

A group of 3264 compounds and 126 targets (in dataset VSD3) was taken for benchmarking. Each compound was used for gathering predictions from each of the 17 publicly accessible target-centric models from web tools (WTCM). Then, the target profile (prediction results) was gathered using scraping techniques.

Table SM2.6: Percentage target' predictions from compounds collected during the scraping process with the target-centric web tools (WTCM) in VSD3.

Web tool algorithm	Collected data (%)
Moltarpred [2]	89.3
Swisstarget Prediction [3]	97.5
Targetnet Scbdd ECFP6 [4]	100
Targetnet Scbdd FP2 [4]	100
Targetnet Scbdd Daylight [4]	100
Targetnet Scbdd MAACs [4]	100
Targetnet Scbdd ECFP2 [4]	100
Targetnet Scbdd ECFP4 [4]	100
Sea Bkslab [5]	98
Extended Connectivity fingerprint ECfp4 NN [6]	91
Shape and Pharmacophore fingerprint Xfp NN [6]	89
Molecular Quantum Numbers MQN NN [6]	87.3
Extended Connectivity fingerprint ECfp4 NNNB [6]	88.5
Shape and Pharmacophore fingerprint Xfp NNNB [6]	89
Molecular Quantum Numbers MQN NNNB [6]	87.3
Extended Connectivity fingerprint ECfp4 NB [6]	88.1
Extended Connectivity fingerprint DNN [6]	69.5

Each web tool has a different size target profile for a compound prediction.

Table SM2.6 shows the percentage of target' predictions from compounds that could be collected. Sometimes, the target profile od a compound could not be gather with an specific model for different external factor like: the non-existence of information because the compound was outside the application domain of the model, the website that hosted the algorithm was down, or an slow response time among others.

Outcome SM2.3. Comparing TCM and WTCM

A set of 3264 compounds in VDS3 were used for comparing both approaches, TCM and WTCM (see methodology detail in section 2.3 in the main manuscript). First, the 15 TCM models were evaluated were terms of TPR, TNR, FPR, FNR; as well as, the recovery and unknown rate (see Table SM2.7). The process was perform later with the WTCM (see Table SM2.8) and the same group of compound-target interactions.

Table SM2.7: Performance of the target-centric models (TCM) in VDS3.

Algorithms	Acronym	TPR	TNR	FPR	FNR	Recovery	Unknown
Decision Tree with FGP	FGP_DT	0.66 ± 0.44	0.48 ± 0.47	0.52 ± 0.47	0.34 ± 0.44	0.98 ± 0.1	0.98 ± 0.01
Gaussian Naive Bayes with FGP	FGP_GM	0.77 ± 0.4	0.47 ± 0.48	0.53 ± 0.48	0.23 ± 0.4	0.98 ± 0.11	0.99 ± 0.01
K-nearest neighbors with FGP	FGP_KNN	0.49 ± 0.48	0.75 ± 0.41	0.25 ± 0.41	0.51 ± 0.48	0.98 ± 0.11	0.98 ± 0.01
Random Forest with FGP	FGP_RF	0.79 ± 0.38	0.53 ± 0.48	0.47 ± 0.48	0.2 ± 0.38	0.98 ± 0.11	0.99 ± 0.01
Support Vector Machine with FGP	FGP_SVM	0.6 ± 0.47	0.64 ± 0.45	0.36 ± 0.45	0.39 ± 0.47	0.98 ± 0.1	0.99 ± 0.01
Decision Tree with DSC	DSC_DT	0.67 ± 0.45	0.56 ± 0.47	0.44 ± 0.47	0.33 ± 0.45	0.99 ± 0.08	0.99 ± 0.01
Gaussian Naive Bayes with DSC	DSC_GM	0.64 ± 0.46	0.61 ± 0.46	0.39 ± 0.46	0.36 ± 0.46	0.99 ± 0.08	0.99 ± 0.01
K-nearest neighbors with DSC	DSC_KNN	0.72 ± 0.43	0.54 ± 0.48	0.46 ± 0.48	0.28 ± 0.43	0.98 ± 0.09	0.99 ± 0.01
Random Forest with DSC	DSC_RF	0.75 ± 0.41	0.61 ± 0.46	0.39 ± 0.46	0.25 ± 0.41	0.98 ± 0.09	0.99 ± 0.01
Support Vector Machine with DSC	DSC_SVM	0.71 ± 0.43	0.64 ± 0.46	0.36 ± 0.46	0.29 ± 0.43	0.98 ± 0.09	0.99 ± 0.01
Decision Tree with FUS	FUS_DT	0.68 ± 0.44	0.54 ± 0.47	0.46 ± 0.47	0.32 ± 0.44	0.98 ± 0.11	0.99 ± 0.01
Gaussian Naive Bayes with FUS	FUS_GM	0.77 ± 0.39	0.5 ± 0.47	0.49 ± 0.47	0.23 ± 0.39	0.98 ± 0.11	0.99 ± 0.01
K-nearest neighbors with FUS	FUS_KNN	0.56 ± 0.47	0.67 ± 0.45	0.33 ± 0.45	0.44 ± 0.47	0.98 ± 0.11	0.99 ± 0.01
Random Forest with FUS	FUS_RF	0.77 ± 0.4	0.56 ± 0.48	0.44 ± 0.48	0.23 ± 0.4	0.98 ± 0.11	0.99 ± 0.01
Support Vector Machine with FUS	FUS_SVM	0.82 ± 0.36	0.51 ± 0.48	$0.48 \pm .48$	0.18 ± 0.36	0.98 ± 0.11	0.99 ± 0.01

TCM were trained with three descriptors: a) Morgan's fingerprint (FGP), b) general physiochemical, structural, and topological molecular properties (DSC), and c) the fusion of both descriptors (FUS).

Table SM2.8: Performance of the target-centric models from web tools (WTCM) in VDS3.

Algorithms	Acronym	TPR	TNR	FPR	FNR	Recovery	Unknown
MolTarPred [2]	MTP	0.69 ± 0.45	0.46 ± 0.49	0.26 ± 0.43	0.29 ± 0.44	0.41 ± 0.48	0.99 ± 0.005
SwissTargetPrediction [3]	STP	0.87 ± 0.32	0.16 ± 0.36	0.75 ± 0.42	0.11 ± 0.23	0.60 ± 0.47	0.99 ± 0.007
Targetnet Scbdd ECFP6 [4]	TS-ECFP6	0.61 ± 0.47	0.5 ± 0.49	0.47 ± 0.49	0.38 ± 0.47	0.6 ± 0.47	0.99 ± 0.01
Targetnet Scbdd FP2 [4]	TS-FP2	0.66 ± 0.46	0.47 ± 0.49	0.5 ± 0.49	0.32 ± 0.45	0.61 ± 0.44	0.99 ± 0.01
Targetnet Scbdd Daylight [4]	TS-Dl	0.56 ± 0.48	0.48 ± 0.49	0.49 ± 0.49	0.42 ± 0.48	0.61 ± 0.44	0.99 ± 0.01
Targetnet Scbdd MACCs [4]	TS-MACCs	0.79 ± 0.39	0.29 ± 0.45	0.67 ± 0.46	0.19 ± 0.38	0.59 ± 0.45	0.99 ± 0.01
Targetnet Scbdd ECFP2 [4]	TS-ECFP2	0.84 ± 0.36	0.24 ± 0.42	0.73 ± 0.44	0.15 ± 0.34	0.61 ± 0.44	0.99 ± 0.01
Targetnet Scbdd ECFP4 [4]	TS-ECFP4	0.69 ± 0.45	0.48 ± 0.49	0.49 ± 0.49	0.29 ± 0.44	0.61 ± 0.44	0.99 ± 0.01
Sea Bkslab [5]	SB	0.99 ± 0.08	0 ± 0	0.74 ± 0.44	0.004 ± 0.06	0.3 ± 0.43	0.99 ± 0.01
Extended Connectivity fingerprint ECfp4 NN [6]	ECfp4-NN	0.99 ± 0.11	NA	0.8 ± 0.39	NA	0.36 ± 0.45	0.99 ± 0.01
Shape and Pharmacophore fingerprint Xfp NN [6]	Xfp-NN	0.98 ± 0.15	NA	0.83 ± 0.38	NA	0.27 ± 0.42	0.99 ± 0.005
Molecular Quantum Numbers MQN NN [6]	MQN-NN	0.98 ± 0.14	NA	0.89 ± 0.31	NA	0.27 ± 0.42	0.99 ± 0.005
Extended Connectivity fingerprint ECfp4 NN + NB1 [6]	ECfp4-NN-NB	0.98 ± 0.14	NA	0.86 ± 0.35	NA	0.38 ± 0.45	0.99 ± 0.006
Shape and Pharmacophore fingerprint Xfp NN + ECfp4 NB [6]	Xfp-NN- ECfp4 -NB	0.98 ± 0.15	NA	0.79 ± 0.41	NA	0.36 ± 0.44	0.99 ± 0.005
Molecular Quantum Numbers MQN NN + ECfp4 NB [6]	MQN-NN- ECfp4 -NB	0.98 ± 0.13	NA	0.83 ± 0.38	NA	0.27 ± 0.42	0.99 ± 0.005
Extended Connectivity fingerprint ECfp4 NB [6]	ECFp4-NB	0.97 ± 0.16	NA	0.78 ± 0.42	NA	0.3 ± 0.43	0.99 ± 0.005
Extended Connectivity fingerprint DNN [6]	ECfp4-DNN	0.98 ± 0.13	NA	0.87 ± 0.34	NA	0.28 ± 0.42	0.99 ± 0.005

Some algorithms simply make predictions about positive compound-target interactions, so the negative predictions are not known along with the TNR and FNR values.

III. Consensus Approach

Note SM2.1. Ensemble Fusion Strategy

Before implementing a consensus strategy with the 15 target-centric models (TCMs), a group of its most representative models is identified. First, a similarity matrix was built using the rand index (RI) metric. Then, a hierarchical clustering dendrogram is generated based on the similarity matrix using the VSD2 and its target profile predictions (see Figure SM2.2a). RI is a well-known score to measure the similarity between two groups [7]. After that, three cutoff values (0.72, 0.75, and 0.8) were used to create clusters of 3, 5, and 7 TCM.

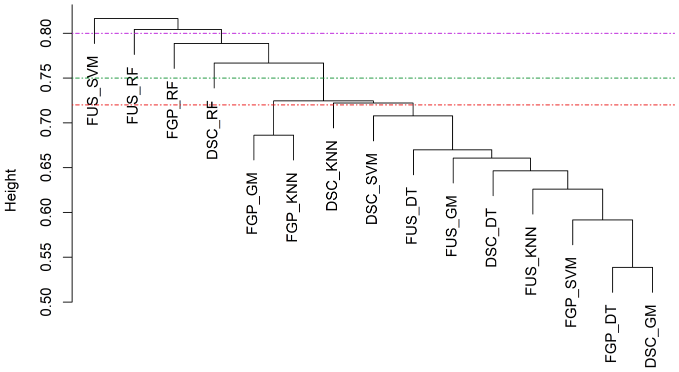

(a) Hierarchical clustering

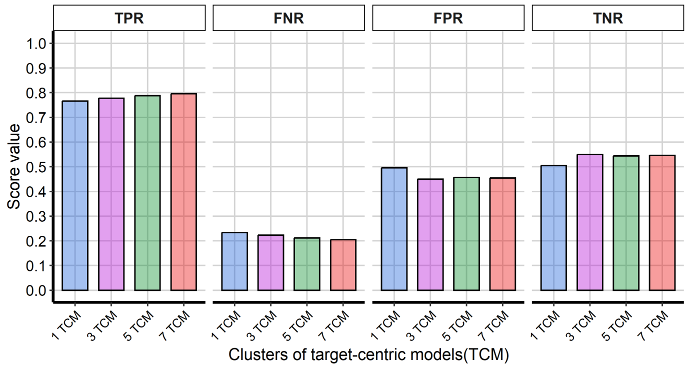

(b) Performance of hierarchical clustering groups

Fig. SM2.2: An ensemble fusion strategy performed in VSD2 to determine clusters that are representative across the 15 TCMs. a) TCM hierarchical clustering with the cutoff values of 0.72, 0.75 and 0.8. b) Clustering performance with the best model (1 TCM) and the three clusters (3,5 and 7 TCM)

Then, to understand the behavior of the grouping several TCM, the model with the highest f1-score (see values on Table SM2.3) is selected from each group to perform the ensemble fusion strategy. he predictions with each model in the cluster was done and the consensus value was computed over VSD2 considering the these clusters: 1 TCM with (FUS_SVM), the 3 TCM' cluster (FUS_GM, FUS_RF, FUS_SVM); the 4 TCM' cluster (FUS_GM, FUS_RF, FUS_SVM, FGP_RF, DSC_RF), and the 7 TCM' cluster (FUS_SVM, FUS_RF, FGP_RF, DSC_RF, FGP_GM, DSC_KNN, FUS_GMM). The output predictions, based on the consensus value, were evaluated with the metrics of true positive rate(TPR), false negative rate(FNR), false positive rate(FPR) and true negative rate (TNR). The cluster results (see Figure SM2.2b and Table SM2.9) indicate a small increment in the performance with 3 TCM. This suggests that these 3 TCM cluster is a good representation of the 15 models, so grouping 3 TCM and performing a consensus based on their predictions could give a more reliable result.

Table SM2.9: Results obtained by the consensus approach with the clusters created with the target centric models (TCM) over VSD2.

Clusters	TPR	FPR	FNR	TNR
1 TCM	0.76	0.49	0.23	0.51
3 TCM	0.77	0.45	0.22	0.55
5 TCM	0.78	0.45	0.21	0.54
7 TCM	0.79	0.45	0.2	0.55

Outcome SM2.4. TCM and WTCM consensus

Results of compound predictions over TCM and WTCM consensus strategy over VSD3 are presented in Table SM2.10 and Table SM2.11 respectively .

Table SM2.10: Results obtained by the consensus approach with the fusion of the three representative target centric models (TCM) over VSD3 across different splits.

Percentage of targets (%)	TPR	FNR	FPR	TNR	Unknown	Recovery
1	0.99	0	0.27	0	0.98	0.08
5	0.99	0	0.42	0	0.87	0.19
10	0.98	0	0.5	0	0.79	0.26
15	0.98	0	0.53	0	0.74	0.31
20	0.98	0	0.68	0	0.68	0.56
50	0.97	0	0.68	0	0.47	0.57
60	0.87	0.01	0.71	0.03	0.39	0.63
70	0.94	0.04	0.68	0.12	0.31	0.71
80	0.87	0.11	0.61	0.28	0.18	0.83
90	0.79	0.38	0.47	0.46	0.06	0.21
100	0.77	0.22	0.45	0.54	0	0.99

Table SM2.11: Results obtained by the consensus approach with the fusion of the 17 target centric models from web tools (WTCM) over VSD3 across different splits.

Percentage of targets (%)	TPR	FNR	FPR	TNR	Unknown	Recovery
1	0.97	0	0.77	0.02	0.56	0.52
5	0.88	0.09	0.69	0.18	0.37	0.68
10	0.81	0.17	0.58	0.29	0.28	0.76
15	0.77	0.22	0.54	0.37	0.22	0.81
20	0.75	0.23	0.52	0.4	0.19	0.83
50	0.7	0.29	0.47	0.51	0.09	0.91
60	0.7	0.29	0.45	0.52	0.08	0.92
70	0.69	0.31	0.44	0.53	0.06	0.94
80	0.68	0.31	0.43	0.54	0.05	0.95
90	0.68	0.31	0.42	0.55	0.04	0.96
100	0.67	0.31	0.42	0.55	0.04	0.96

IV. Web tool

Note SM2.2. Web-tool implementation

A web tool is created for target identification with the trained target-centric models (TCM) and its consensus approach with a simple and intuitive interface at https://bioquimio.udla.edu.ec/tidentification01/. The TCM are built with the algorithms of decision tree (DT), gaussian naive bayes (GM), k-nearest neighbors (KNN), random forest (RF), and support vector machine (SVM), considering three groups of descriptors: i) morgan's fingerprint (FGP), ii) general molecular properties (DSC), and iii) the fusion of both descriptors (FUS).

The web tool utility was made available without requiring a login. It has an input field for smiles of query compounds, and four tabs for the consensus approach and for each group of descriptors: FGP, DSC, and FUS (Figure SM2.3a). If the smile is written wrong, there is also a button to clear the input field. The backend is implemented using the *Flask Python framework* [1] and the frontend is built in PHP, JavaScript, HTML and css. Also, an example with a help page is provided to make its use easier. The compound-target predictions can be run by pasting the compound's query as smiles and clicking the submit button. The smiles are checked to ensure that they conform to valid structures and are within the applicability domain of each model before the prediction is performed.

Then, the query compound is drawn (Figure SM2.3b) and the projected target profile is given in a table when the calculation is complete (Figure SM2.3c). The output target profile includes a list of targets with the predicted interaction [0,1] activity for each TCM model. The target profile also includes the probability of having a positive interaction with the target next to the binary class in parenthesis. In addition, a searcher is shown upper the target profile to facilitate the query of the targets. Each target contains the UniProt ID and a hyperlink to the UniProt database. UniProt is a

[1] Flask(https://flask.palletsprojects.com/en/2.3.x/) is a micro web framework that has useful tools and features for developing RESTful APIs.

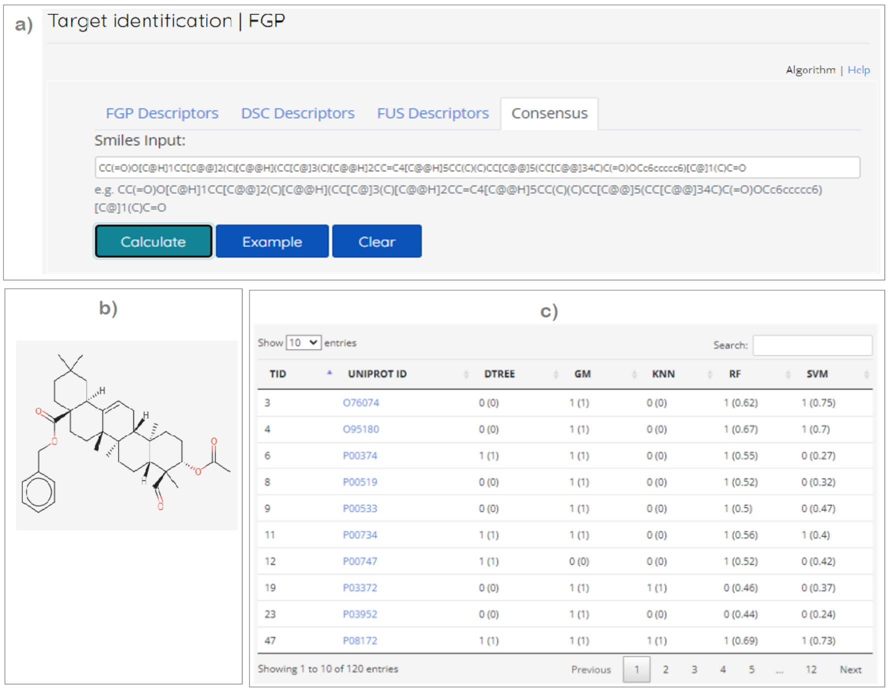

Fig. SM2.3: Target identification web tool a) Form to put the query molecule considering the required descriptors, b) Query molecule graph. c) Output list of predicted target interactions.

comprehensive and freely accessible database of protein sequences and functional information [8]. It is maintained by the UniProt consortium and contains a large amount of information on protein biological functions drawn from the research literature.

References

[1] Nanda, A., Mahapatra, A., Mohapatra, B., mahapatra, a.: Multiple comparison test by tukey's honestly significant difference (hsd): Do the confident level control type i error. International Journal of Applied Mathematics and Statistics **6**, 59–65 (2021) https://doi.org/10.22271/maths.2021.v6.i1a.636

[2] Peón, A., Li, H., Ghislat, G., Leung, K., Wong, M.H., Lu, G., Ballester, P.: Moltarpred: A web tool for comprehensive target prediction with reliability estimation. Chemical Biology amd Drug Design **94**, 1390–1401 (2019) https://doi.org/10.1111/cbdd.13516

[3] Daina, A., Michielin, O., Zoete, V.: Swisstargetprediction: updated data and new features for efficient prediction of protein targets of small molecules. Nucleic Acids Research **47**(W1), 357–364 (2019) https://doi.org/10.1093/nar/gkz382

https://academic.oup.com/nar/article-pdf/47/W1/W357/28880175/gkz382.pdf

[4] Yao, Z., Dong, J., Che, Y.-J., Zhu, M.-F., Wen, M., Wang, N., Wang, S., Lu, A., Cao, D.-S.: Targetnet: a web service for predicting potential drug–target interaction profiling via multi-target sar models. Journal of Computer-Aided Molecular Design **30** (2016) https://doi.org/10.1007/s10822-016-9915-2

[5] Keiser, M., Roth, B., Armbruster, B., Ernsberger, P., Irwin, J., Shoichet, B.: Relating protein pharmacology by ligand chemistry. Nature biotechnology **25**, 197–206 (2007) https://doi.org/10.1038/nbt1284

[6] Awale, M., Reymond, J.-L.: The polypharmacology browser ppb2: Target prediction combining nearest neighbors with machine learning. Journal of Chemical Information and Modeling **59** (2018) https://doi.org/10.1021/acs.jcim.8b00524

[7] Al-amri, R., Murugesan, R.K., Almutairi, M., Munir, K., Alkawsi, G., Baashar, Y.: A clustering algorithm for evolving data streams using temporal spatial hyper cube. Applied Sciences **12**(13) (2022) https://doi.org/10.3390/app12136523

[8] Bateman, A., Martin, M.-J., Orchard, S., Magrane, M., Agivetova, R., Ahmad, S., Alpi, E., Bowler-Barnett, E., Britto, R., Bursteinas, B., Bye-A-Jee, H., Coetzee, R., Cukura, A., Silva, A., Denny, P., Dogan, T., Ebenezer, T., Fan, J., Castro, L., Teodoro, D.: Uniprot: the universal protein knowledgebase in 2021. Nucleic Acids Research **49** (2020) https://doi.org/10.1093/nar/gkaa1100
